# Integrated transcription factor profiling with transcriptome analysis identifies L1PA2 transposons as global regulatory modulators in a breast cancer model

**DOI:** 10.1038/s41598-021-86395-9

**Published:** 2021-04-13

**Authors:** Jiayue-Clara Jiang, Joseph A. Rothnagel, Kyle R. Upton

**Affiliations:** School of Chemistry and Molecular Biosciences, The University of Queensland, St Lucia, QLD 4072 Australia

**Keywords:** Breast cancer, Cancer genetics, Cancer genomics, Oncogenes, Gene regulatory networks, Genome informatics, Epigenetics, Transcriptomics, Cancer genetics, Cancer genomics, Epigenetics, Gene regulation, Genomics

## Abstract

While transposons are generally silenced in somatic tissues, many transposons escape epigenetic repression in epithelial cancers, become transcriptionally active and contribute to the regulation of human gene expression. We have developed a bioinformatic pipeline for the integrated analysis of transcription factor binding and transcriptomic data to identify transposon-derived promoters that are activated in specific diseases and developmental states. We applied this pipeline to a breast cancer model, and found that the L1PA2 transposon subfamily contributes abundant regulatory sequences to co-ordinated transcriptional regulation in breast cancer. Transcription factor profiling demonstrates that over 27% of L1PA2 transposons harbour co-localised binding sites of functionally interacting, cancer-associated transcription factors in MCF7 cells, a cell line used to model breast cancer. Transcriptomic analysis reveals that L1PA2 transposons also contribute transcription start sites to up-regulated transcripts in MCF7 cells, including some transcripts with established oncogenic properties. In addition, we verified the utility of our pipeline on other transposon subfamilies, as well as on leukemia and lung carcinoma cell lines. We demonstrate that the normally quiescent regulatory activities of transposons can be activated and alter the cancer transcriptome. In particular, the L1PA2 subfamily contributes abundant regulatory sequences, and likely plays a global role in modulating breast cancer transcriptional regulation. Understanding the regulatory impact of L1PA2 on breast cancer genomes provides additional insights into cancer genome regulation, and may provide novel biomarkers for disease diagnosis, prognosis and therapy.

## Introduction

Transposons are repetitive DNA elements that are ubiquitous in eukaryotic genomes, and occupy about 45% of the human genome^1^. Transposons contain cis-regulatory sequences that are recognised by the host transcriptional machinery (primarily transcription factors (TF) and RNA polymerases (RNA pol)), which they require to exploit the host transcriptional resources for their own replication^2–7^. This obligatory compatibility also enables the host genome to exapt transposon-derived regulatory sequences to modulate the regulation of host transcriptional networks.

Transposons have been shown to contribute functional sequences, including TF binding sites (TFBS), promoters, enhancers and insulators, for the regulation of host genes in a variety of organisms, including humans, mice, maize and fruit flies^2,8–13^. Host genes that are under the regulation of transposon sequences are involved in a diverse range of biological functions, including innate immunity and pregnancy in humans, female fertility and brain development in mice, as well as courtship song phenotype and stress response in fruit flies^2,8–12^.

In addition to gene regulation in normal biological pathways, transposons may also play a regulatory role in the cancer transcriptome. In the human genome, transposons are often heavily suppressed in somatic tissues, primarily via histone tail modifications and DNA methylation; however, they can escape epigenetic repression in the cancer state, and exert regulatory activity that affects the expression of oncogenes^14–17^. The transposon-derived activation of oncogenes, called the onco-exaptation of transposons, can subsequently lead to tumour development and enhanced malignancy, as summarised in Babaian and Mager^17^. Specific examples of transposon-driven abnormal oncogene expression have been reported in human lymphoma, leukemia, lung cancer, bladder cancer, and breast cancer^18–26^.

In 1971, Britten and Davidson proposed that the expansion of transposons in the host genome could have accelerated the evolution of regulatory networks, by acting as a vector to disperse regulatory elements and thereby recruiting host genes into co-regulated, co-expressed networks^27^. Based on this theory, it is expected that regulatory functions, as well as features that account for regulatory activities will be conserved within a group of related transposons. Indeed, transposons within the same subfamilies have been demonstrated to exhibit similar regulatory activities. For example, in silico analysis has revealed the MER130 DNA transposon subfamily to be significantly enriched within active enhancers in the mouse dorsal cerebral wall^10^. These MER130 transposons contain a highly conserved region harbouring putative binding motifs for TFs associated with brain development, and exhibit in vitro enhancer activity in mouse embryonic neurons^10^. In human innate immunity, LTR retrotransposons are overrepresented in the transcriptional regulatory network^2^. In particular, upon interferon-gamma stimulation, the MER41 subfamily is significantly enriched in the binding sites of IRF1 and STAT1, both of which play a crucial role in immune signalling pathways^2^. Several MER41 transposons also contribute enhancer activity for immunity-associated genes, such as *AIM2* and *APOL1*^2^. In the case of pregnancy, the MER20 subfamily has been found to harbour binding sites for hormone-responsive and pregnancy-related TFs, and are overrepresented near progesterone or cAMP-responsive endometrial genes^28^. Epigenetic profiling indicates that these MER20 transposons can exert a diverse range of regulatory activities by acting as enhancers, insulators or repressors^28^. As demonstrated by examples above, transposons can exert regulatory activity at the subfamily level, and this conservation of regulatory activity allows functionally correlated genes to be regulated in a temporally or spatially specific manner.

**Figure 1:**
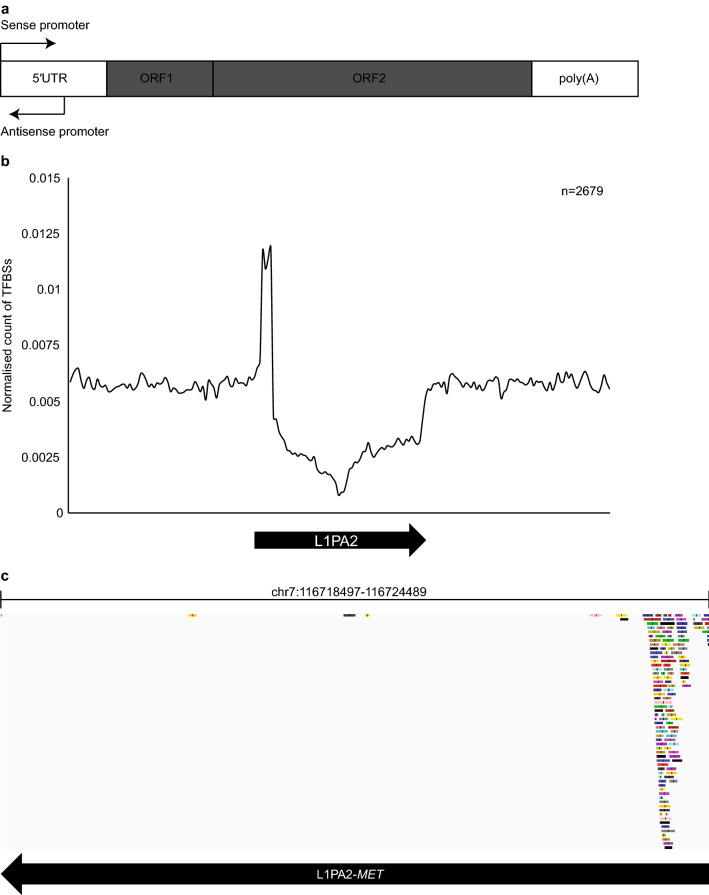
Distribution of TFBSs in human L1PA2 transposons and surrounding regions. (**a**) A typical LINE1 transposon, approximately 6 kb in length, contains a 5′ UTR, two ORFs and a 3′ poly(A) tail^1,29,30^. (**b**) The 5′ UTR of L1PA2 was a prominent source of TFBSs. The normalised counts of TFBSs in the 20 kb region (bin = 100 bp) centred on L1PA2 transposons are shown, averaged by the total number of L1PA2 harbouring at least one GTRD meta cluster (n = 2,679). While the majority of L1PA2 structures showed a depletion of TFBSs, the 5′ UTR was a prominent reservoir of TFBSs. (**c**) Example GTRD browser view of TFBS distribution in L1PA2 transposons. The TF binding distribution in the L1PA2 transposon located upstream of the *MET* gene was visualised on the GTRD browser^39^. Each rectangular track indicates a TF binding meta-cluster. The black arrow indicates the position and orientation (5′ to 3′) of the L1PA2 transposon.

LINE1 retrotransposons are abundant in the human genome, with over 500,000 copies occupying 17% of the genomic sequence^1^. A full-length LINE1 element is approximately 6 kb in length, and contains a 5′ untranslated region (5′ UTR), two open reading frames (ORF) and a 3′ poly(A) tail^1^ (Fig. 1a). The 5′ UTR of LINE1 transposons harbours a bi-directional promoter^29,30^ (Fig. 1a). LINE1 transposons are classified into subfamilies by the presence of ancestral or post-insertion mutations in their sequences^31^. L1PA2 transposons are a primate-specific subfamily of LINE1 elements, with approximately 4,940 copies in the human genome^32^. According to RepeatMasker annotations^33,34^, 978 out of 4,940 (19.8%) human L1PA2 elements are over 6 kb in length, exhibiting limited divergence, deletion and insertion relative to the consensus sequence (see Supplementary Fig. S1 online).

We previously reported the regulatory activity of several transposon subfamilies in the context of breast cancer^35^. Our analysis revealed that the L1PA2 transposons were significantly enriched in E2F1 and MYC binding sites in MCF7 cells. Furthermore, luciferase assays in triple negative breast cancer cells confirmed the activity of an L1PA2-derived promoter, where the transposon was found to account for a significant proportion of promoter activity to the *SYT1* gene^35^. In support of our findings, a recent paper by Jang et al. showed that the L1PA2-derived promoter activity was not limited to *SYT1*, and was also seen for other oncogenes, such as *MET, XCL1* and *AKAP13*, in a number of cancer types^22–25,36^. In particular, the L1PA2*-MET* alternative transcript has been found in chronic myeloid leukemia, colorectal cancer and breast cancer, and is correlated with enhanced metastasis and poor prognosis^22–25^. Intrigued by our results and the literature evidence, we aimed to conduct an integrated investigation on the regulatory activity of the L1PA2 subfamily in breast cancer cells.

In this study, we have developed a bioinformatic pipeline for the integrated analysis of transcription factor binding and transcriptomic data to identify transposon-derived promoters that are activated in specific diseases and developmental states. Using this pipeline, we showed that the L1PA2 transposon subfamily contributed abundant regulatory sequences to co-ordinated transcriptional regulation in breast cancer. Over 27% of L1PA2 transposons contained at least one TFBS in MCF7 breast cancer cells, and the binding of TFs was correlated with active epigenetic modifications. Upon further investigation, several TFs were found to show co-localised binding sites in L1PA2, supporting our hypothesis that L1PA2 could serve as a vector for dispersing functionally related regulatory sequences. These L1PA2-binding TFs formed highly interacting networks, which were functionally enriched for transcriptional mis-regulation in a variety of cancer types. L1PA2 transposons also constituted an abundant reservoir of transcription start sites (TSS) in MCF7 cells. These L1PA2 transposons displayed an active epigenetic profile in MCF7 cells, and contributed cancer-specific promoter activity to a number of alternative or novel transcripts. Our results demonstrate that the normally quiescent regulatory activities of transposons can be unleashed following the loss of epigenetic repression, altering the cancer transcriptome. Taken together, the ubiquitous and replicative nature of the L1PA2 subfamily makes them an exemplary vector for the dispersal of co-localised transcription factor binding sites, thereby facilitating the co-ordinated regulation of genes. We demonstrate that the L1PA2 subfamily is a prominent contributor of regulatory elements in breast cancer cells, as well as leukemia and lung cancer cells, and likely play a global role in cancer transcriptional regulation. The transcriptional activation of L1PA2 transposons in cancer is correlated with alterations in the transcriptome, and may provide novel biomarkers for disease diagnosis and treatment.

## Results

### The L1PA2 5′ UTR is a rich reservoir of human TFBSs

To investigate the pattern of TF binding in L1PA2 elements, the occurrence of TFBSs within L1PA2 transposons and neighbouring regions were counted. 2,679 out of 4,940 (54.2%) L1PA2 transposons harboured at least one TFBS when all experimental conditions (tissues, cell lines, treatments etc.) were considered. The 5′ UTR of L1PA2 transposons harboured abundant TFBSs, as the frequency of TF binding almost doubled in the 5′ UTR compared to neighbouring genomic regions (Fig. 1b). In contrast, the middle of the transposon sequence had a notable depletion (Fig. 1b). This region contains the coding sequences for the LINE1 machinery, and this depletion is consistent with depletion of TF binding within exons^37^. Decreased mappability within these regions may also have some effects on the appearance of depletion compared to surrounding genomic regions^38^. An example of TF binding, as visualised on the GTRD browser^39^, is shown for the L1PA2 transposon located upstream of *MET*, previously described by Roman-Gomez, et al.^22^ (Fig. 1c).

### Over 27% of L1PA2 transposons harbour TFBSs in a breast cancer cell line

Next we analysed cell-type-specific ChIP-seq data from the ChIP-Atlas database to investigate the TF binding status of L1PA2 transposons in the context of breast cancer^40^. The ChIP-Atlas dataset represents TFBSs identified in MCF7 cells subjected to a diverse range of experimental conditions, including hormone treatments with estradiol and progesterone^40^. A total of 1,376 out of 4,940 (27.9%) L1PA2 elements harboured at least one TFBS in MCF7 breast cancer cells alone.

**Figure 2:**
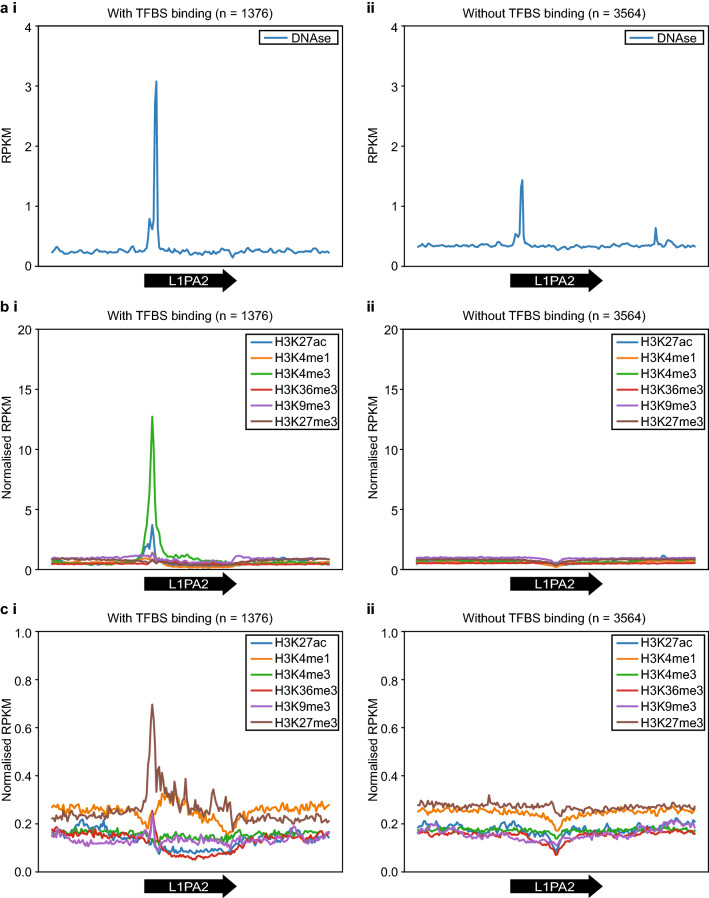
TF binding in L1PA2 was correlated with active epigenetic marks in MCF7 cells. In MCF7 cells, L1PA2 transposons bound by TFs were associated with (**a**) increased DNAse sensitivity (**b**) active histone tail modifications. (**c**) However, in the normal mammary tissues, TF binding in L1PA2 transposons was counteracted by repressive histone tail modifications. The average RPKM values of the DNAse-seq data and the normalised RPKM values of histone modification ChIP-seq data are shown for the 20 kb region (bin = 100 bp) centred on L1PA2 transposons (**i**) with, and (**ii**) without TFBSs. Average RPKM values were calculated by averaging the raw RPKM values of each epigenetic mark across the replicates. Normalised RPKM values were calculated by normalising the average RPKM values to the control ChIP-seq data by subtraction. Black arrows indicate the position and orientation (5′ to 3′) of L1PA2 transposons.

To investigate whether this binding was the result of functional or spurious binding events, we investigated the DNAse sensitivity of L1PA2 transposons via the analysis of DNAse-seq datasets. DNAse hypersensitivity assays provide a proxy for open chromatin regions, but do not necessarily indicate active transcription^41,42^. However, there is a general positive correlation between DNAse signal and transcriptional activity^43^. Interestingly, DNAse hypersensitivity was observed in the 5′ UTR of both bound and unbound elements; however, the strength of this signal was around twice as strong for bound elements, supporting a more open chromatin configuration and transcriptional activity from bound transposons (Fig. 2a and Supplementary Fig. S2 online).

To further confirm this apparent epigenetic activation associated with TF binding, we investigated the histone states of L1PA2 elements in MCF7 cells by analysing publicly available ChIP-seq datasets. We reasoned that functional binding would be associated with active chromatin marks commonly observed in promoters and enhancers (H3K27ac, H3K4me1, and H3K4me3), or gene bodies (H3K36me3), but not repressive chromatin marks (H3K9me3 and H3K27me3)^44–46^. We found that active histone modifications were enriched in the 5′ UTR of TF-bound L1PA2 elements, indicating these elements were transcriptionally active (H3K27ac), with a strong promoter profile (H3K4me3), and some elements potentially contained primed enhancer properties (H3K4me1) (Fig. 2b and Supplementary Fig. S2 online). No notable features were observed for H3K36me3 histone modifications that usually mark active gene bodies or repressive histone marks (Fig. 2b and Supplementary Fig. S2 online). By contrast, in the normal mammary tissues, TF-bound L1PA2 elements were associated with a notable increase in the repressive histone modifications, particularly H3K27me3 (Fig. 2c). Repressive histone modifications were not observed in the absence of TF binding in either cancer or normal conditions, suggesting that repression via histone modifications acts as a counteracting force to maintain transposon repression in the normal state. This force would not be required, and is not observed, in the absence of TF binding in either the normal or cancer state. Taken together, this data strongly supports the existence of L1PA2-derived promoters and potentially L1PA2-derived enhancer elements in MCF7 breast cancer cells.

### L1PA2 contains co-localising TFBSs in MCF7 cells

**Figure 3:**
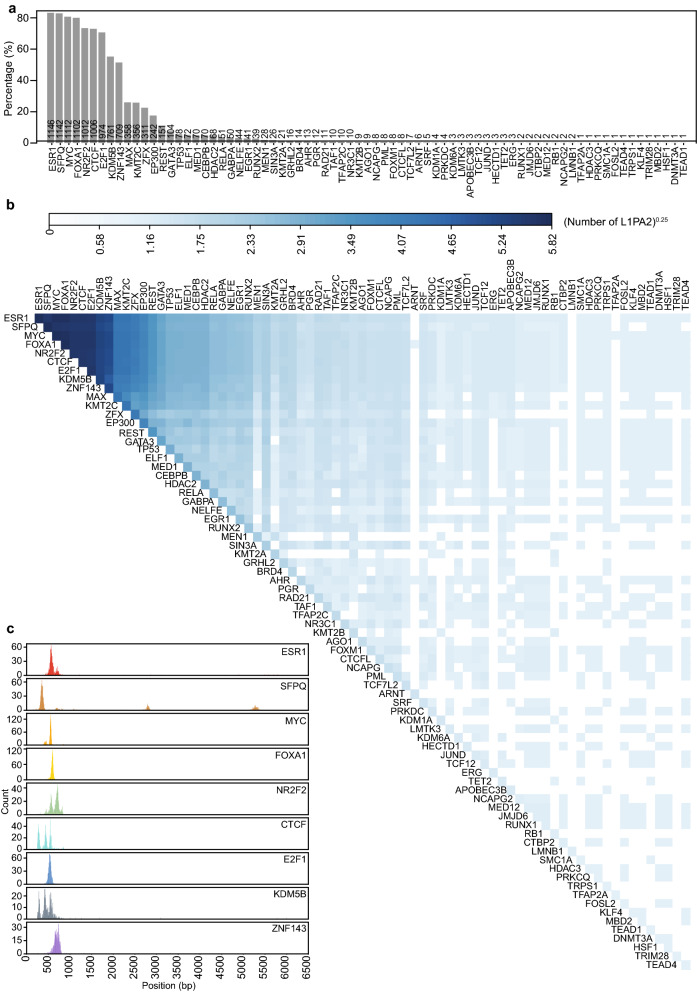
L1PA2 contained co-localising TFBSs in MCF7 cells. (**a**) A total of 75 TFs were found to bind to L1PA2 transposons in MCF7 cells. The bars and numbers show the percentages and counts of TF-bound L1PA2 transposons (n = 1,376) respectively. (**b**) For each pair of TFs, the number of L1PA2 transposons containing both TFBSs is shown in the heatmap, where the counts are transformed (n^0.25^). (**c**) The majority of TFBSs co-localised to the 5′ UTR of L1PA2s. Histograms indicate the distribution of TFBSs for the most frequently binding TFs in the consensus L1PA2 transposon (bin = 5 bp).

A total of 75 TFs, including ESR1, SFPQ and MYC, were found to bind to L1PA2 transposons in MCF7 cells (Fig. 3a). We sought to investigate whether these TFs were known to bind in a co-ordinated manner, which is an essential feature of transcriptional complexes^47,48^. We confirmed that in MCF7 cells, a number of L1PA2 transposons showed a consistent TF binding pattern where they were found to harbour co-localised TFBSs (Fig. 3b). More specifically, binding sites of TFs with the highest binding frequency (ESR1, SFPQ, MYC, FOXA1, NR2F2, CTCF, E2F1, KDM5B and ZNF143) predominantly mapped to the 5′ UTR of L1PA2s, and these binding sites often occurred within close proximity to each other (Fig. 3c and Supplementary Fig. S3 online). The TF binding profile in MCF7 cells appeared to be subfamily-specific, as other transposon subfamilies showed a different signature of TF binding and that closely related LINE1 subfamilies exhibited similar TF binding signatures (Supplementary Fig. S4 online).

### TFBS co-localisation occurs more often in L1PA2 compared to the rest of the genome

**Figure 4:**
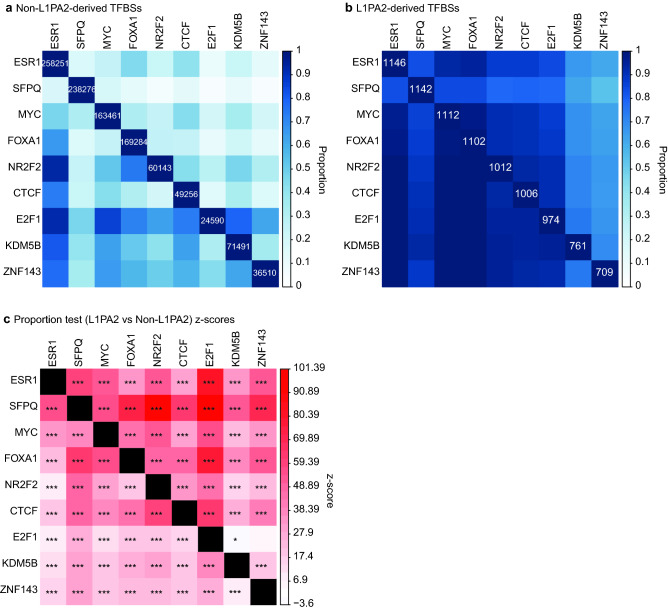
Top binding TFs co-occurred more frequently in L1PA2s than in the rest of the genome. The proportions of co-occurrence are shown for (**a**) non-L1PA2-derived and (**b**) L1PA2-derived TFBSs. For each TF, the total number of binding sites is shown in the diagonal. (**c**) For each TF-TF combination, the co-localisation proportions were compared (L1PA2 versus non-L1PA2) using a one-tailed proportion z-test. The heatmap indicates the z-scores of the proportion tests, where a positive z-score (red) indicates more co-localisation in L1PA2s, and a negative z-score (blue) indicates more co-localisation in the rest of the genome. P < 6.2E-4: *, p < 0.0001: **, p < 0.00001: ***. To account for asymmetrical co-localisation, the proportions and z-scores correspond to the chances of the TF in the row co-localising with the TF in the column.

We next compared the occurrence of TFBS co-localisation in L1PA2 transposons and genome-wide. For each pair of frequently binding TFs, the proportions of TFBSs co-occurring with each other were calculated with each factor as the denominator separately to account for asymmetrical co-localisation, which occurs as a function of different total binding site counts for individual TFs. For example, considering genome-wide TFBSs, while nearly all NR2F2 binding sites (93%) co-localised with ESR1 binding sites, only 23.2% of ESR1 sites co-localised with NR2F2 sites (Fig. 4a). This asymmetry can be seen as a function differences in the total number of binding sites for each factor, where ESR1 binding sites were four times more abundant than NR2F2 binding sites.

Overall, asymmetrical co-localisation was common when considering genome-wide TFBSs (Fig. 4a). In contrast, the total numbers of L1PA2-derived TFBSs were more similar amongst the top binding TFs, which led to less asymmetrical co-localisation, and supported the hypothesis that these TFBSs co-occurred more in L1PA2 transposons (Fig. 4b). Indeed, proportion tests revealed that, overall, the binding sites of top binding TFs co-occurred more frequently in L1PA2 transposons when compared to the rest of the genome (Fig. 4c). The only exceptions involved E2F1, where it appeared to co-localise with KDM5B less frequently in L1PA2, and displayed no significant enrichment or depletion for ZNF143 co-localisation. Compared to the other transposon subfamilies, L1PA2 transposons showed the highest degree of binding site co-localisation (Supplementary Fig. S4 online), further confirming their prominent role in modulating the co-ordinated gene regulation in breast cancer transcriptome.

### L1PA2 transposons harbour less TF binding activity in MCF10A near-normal cells

**Figure 5:**
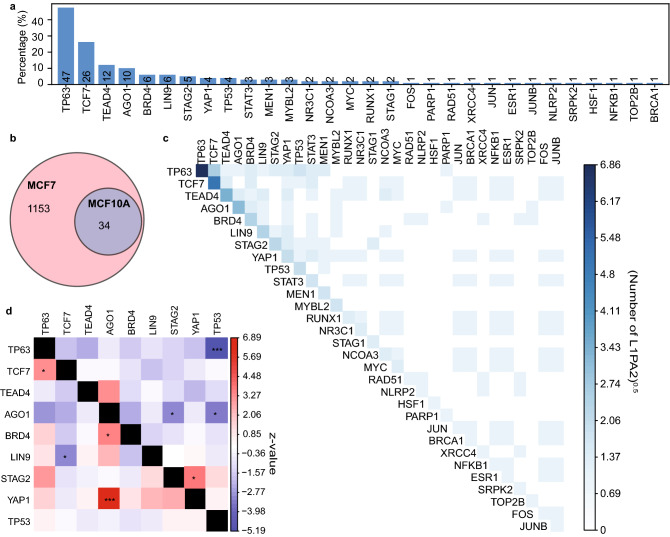
L1PA2 transposons showed fewer occurrences of TF binding and binding site co-localisation in MCF10A near-normal cells. (**a**) A total of 30 TFs were found to bind L1PA2 transposons in MCF10A cells. The bars and numbers show the percentages and counts of TF-bound L1PA2 transposons (n = 99) respectively. (**b**) The venn diagram shows the overlaps between L1PA2 transposons that harbour TFBSs in MCF7 and MCF10A cells, considering only TFs that were studied in both cell lines (not drawn to scale). (**c**) TFBS co-localisation for frequently binding TFs in MCF10A cells. For each pair of TFs, the number of L1PA2 transposons containing both TFBSs in MCF10A cells is shown in the heatmap, where the counts are transformed (n^0.25^). (**d**) Co-localisation enrichment analysis was performed for the most frequently binding TFs. For each TF-TF combination, the co-localisation proportions were compared (L1PA2 versus non-L1PA2) using a one-tailed proportion z-test. The heatmap indicates the z-scores of the proportion tests, where a positive z-score (red) indicates more co-localisation in L1PA2s, and a negative z-score (blue) indicates more co-localisation in the rest of the genome. P < 6.2E−4: *, p < 0.0001: **, p < 0.00001: ***. To account for asymmetrical co-localisation, the proportions and z-scores correspond to the chances of the TF in the row co-localising with the TF in the column.

To test our hypothesis that L1PA2 transposons contributed increased regulatory activity in the cancer state, we also investigated the TF binding profile of L1PA2 transposons in the near-normal MCF10A cell line. Overall, a total of 30 TFs were identified to have binding sites within L1PA2 transposons in MCF10A cells (Fig. 5a). It is worth noting that fewer TFs had been studied in MCF10A cells (41 TFs in MCF10A versus 100 TFs in MCF7) based on the ChIP-Atlas collection, and this asymmetry in data availability limits direct comparison between cell lines. Nevertheless, a set of 16 TFs, including important regulators such as ESR1 and TP53, have been studied in both cell lines and were used to directly compare L1PA2 binding in MCF7 and MCF10A cells. Only 34 L1PA2 elements were bound in MCF10A cells, all of which were also bound in MCF7 cells (Fig. 5b), while an additional 1,153 L1PA2 elements were bound in MCF7 cells exclusively. This reduced TF binding in MCF10A cells coincided with the lack of active epigenetic marks in the normal mammary tissues (Figs. 2c and 5b). Furthermore, L1PA2 transposons in MCF10A displayed a notably different TFBS co-localisation pattern, confirming that L1PA2-derived binding site co-localisation was more prominent in the cancer state (Fig. 5c,d).

### L1PA2 onco-exaptation is a common event in cancer

We next sought to determine whether the transcriptional activation of L1PA2 transposons was a common feature in cancer, by investigating their TF binding activity in other cancer types. We characterised L1PA2-derived TFBSs in K562 and A549 cells, which are leukemia and lung carcinoma cell lines respectively. Similar to MCF7 cells, L1PA2 transposons were found to contribute abundant TF binding activity in the other cancer cell lines. In particular, 2,598 (52.6%) and 1,121 (22.7%) out of 4,940 L1PA2 transposons were found to contain TFBSs in K562 and A549 cells respectively. Upon further examination, 1,096 L1PA2 elements harboured TF binding activity in all three cancer cell lines, suggesting that the transcriptional activation and onco-exaptation of the L1PA2 subfamily was a common phenomenon in cancer (Supplementary Fig. S5 online).

### L1PA2 contains binding sites for highly interacting, cancer-associated TF networks in MCF7 cells

Many TFs, such as FOXA1 and ESR1, have been shown to function in a context-specific manner, dependent on specific protein–protein interactions, resulting in pro-oncogenic properties in some cancer types, and anti-oncogenic properties in others^49,50^. We therefore interrogated known biological observations of protein–protein interactions, and performed functional enrichment analysis to identify biological functions and disease states associated with the L1PA2-binding TFs.

**Figure 6:**
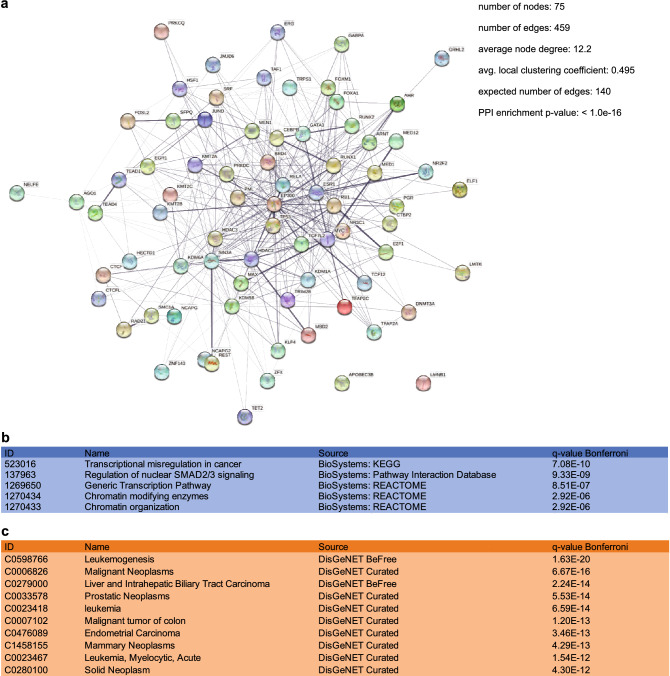
L1PA2 contained binding sites for highly interacting, cancer-associated TF networks in MCF7 cells. (**a**) L1PA2-binding TFs were predicted to form highly interacting networks according to STRING analysis^51^. Edges represent protein–protein interactions with experimental evidence, and the thickness of edges represents level of confidence. Statistical analysis results from STRING are shown. (**b**) Pathway enrichment analysis by ToppFun showed that L1PA2-binding TFs were enriched for transcriptional mis-regulation in cancer^52^. Top enriched pathways are shown. (**c**) Disease enrichment analysis by ToppFun showed that L1PA2-binding TFs were enriched in various cancer types^52^. Top enriched diseases are shown.

STRING analysis showed that these 75 TFs formed highly interacting networks, based on available experimental evidence (PPI enrichment p-value < 1E−16)^51^ (Fig. 6a). Functional enrichment analysis by ToppFun revealed that these TFs were significantly enriched in 44 pathways, with the most significant enrichment reported in transcriptional mis-regulation in cancer (Bonferroni q-value = 7.08E−10)^52^ (Fig. 6b). For the complete list of significant pathways see Supplementary Table S1 online.

Considering diseases, ToppFun analysis showed that these TFs were significantly enriched in 228 diseases, 183 of which are neoplastic diseases, including prostate cancer (prostatic neoplasms, ranked 4^th^) (Bonferroni q-value = 5.53E−14) and breast cancer (mammary neoplasms, ranked 8^th^) (Bonferroni q-value = 4.29E−13)^52^ (Fig. 6c). For the complete list of significant diseases see Supplementary Table S2 online.

### Oncogenic TF binding motifs are conserved in the L1PA2 5′ UTR

**Figure 7:**
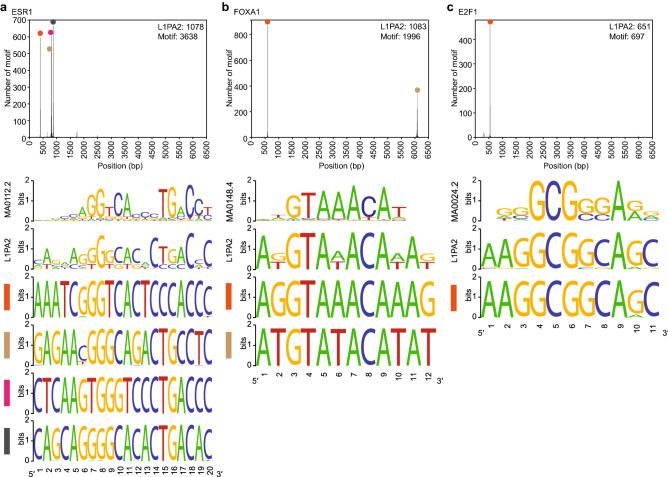
Oncogenic TF binding motifs are conserved in the 5′ UTR of L1PA2 transposons. Motif analysis was performed to identify the binding motifs for (**a**) ESR1, (**b**) FOXA1 and (**c**) E2F1 in L1PA2 transposons. The positions of the discovered motifs in the consensus L1PA2 sequence are shown in the histograms (bin = 5 bp). The number of L1PA2 elements containing at least one statistically significant motif, and the total number of statistically significant motifs are shown. For each TF, the JASPAR sequence logo is shown, labelled with the corresponding JASPAR motif ID. The “L1PA2” sequence logo was generated using all discovered motif sequences. Each peak in the histogram is labelled with a colour, and the sequence logos generated from motifs in the corresponding bins are shown.

We hypothesised that the transcription factor binding activity shared amongst L1PA2 transposons was correlated with conserved motif sequences. The sequence similarity amongst L1PA2 transposons impeded de novo motif discovery (data not shown), we thus performed motif scanning analysis to identify occurrences of known binding motifs of frequently binding, oncogenic TFs (ESR1, FOXA1 and E2F1) in L1PA2 sequences. Overall, 66.8%-98.3% of TF-bound L1PA2 transposons contained at least one significant motif of the corresponding TF (Fig. 7). The majority of binding motifs mapped to the 5′ UTR of L1PA2 elements (Fig. 7). In particular, four ESR1 binding motif peaks were identified in the 5′ UTR, and each peak contained a highly consistent, overrepresented sequence, which was similar to the palindromic estrogen response element. For FOXA1, the majority of the discovered motifs were located in the 5′ UTR, with a small number mapping to the 3′ end of L1PA2 transposons. E2F1 binding motifs showed the highest level of conservation, with highly consistent motifs identified almost exclusively in the 5′ UTR.

### TF binding in L1PA2 transposons is correlated with the activation of nearby genes

The promoter regions of genes, which harbour crucial regulatory sequences, are usually found within 1 kb of the TSSs, while other regulatory elements, such as enhancers, are typically located farther away from their target genes^53^. To investigate the regulatory role of L1PA2-derived TF binding, the distribution of L1PA2 transposons with respect to breast cancer-associated genes was analysed using publicly available RNA-seq data from MCF7 breast cancer and MCF10A near-normal cells. 9,375 and 9,771 transcripts were up-regulated and down-regulated in MCF7 cells respectively, with 5,435 up- and 4,094 down-transcripts passing a p-value cut-off of 0.05 (see Supplementary Tables S3 and S4 online). Overall, TF-bound L1PA2 transposons were correlated with the activation of breast cancer genes in MCF7 cells, as a higher percentage of TF-bound L1PA2 transposons were found in the promoter regions, as well as up to 20 kb away from the TSSs of up-regulated transcripts (see Supplementary Fig. S6 online). In contrast, TF-bound L1PA2 transposons were found less frequently in the promoter or surrounding 20 kb regions of down-regulated transcripts (see Supplementary Fig. S6 online). Similarly, L1PA2 transposons that lacked TF binding were generally absent from the promoter regions of differentially expressed transcripts (see Supplementary Fig. S6 online).

### L1PA2 transposons are overrepresented in the TSSs of up-regulated genes

To assess the contribution of L1PA2 transposons to transcript initiation, direct overlaps between L1PA2 and the TSSs of differentially expressed transcripts were analysed. 41 out of 1,376 TF-bound L1PA2 transposons harboured the TSSs of up-regulated transcripts, while only one L1PA2 harboured the TSS of a down-regulated transcript. To test whether the correlation between TSSs and L1PA2 transposons was due to random chance, enrichment analysis by random rotation of the genome was performed, and showed that L1PA2 transposons were significantly enriched for the TSSs of up-regulated transcripts (p = 4.43E−11), and significantly depleted for down-regulated transcripts (p = 2.55E−09) (Supplementary Table S5 online). In contrast, other transposon subfamilies showed the opposite pattern, where they were significantly depleted for the TSSs of up-regulated transcripts (Supplementary Table S5 online). L1PA2 transposons harbouring TSSs of up-regulated transcripts are summarised in Supplementary Table S6 online. For results of transcript expression quantification by StringTie^54^ see Supplementary Fig. S7 online.

### L1PA2 transposons bearing up-regulated TSSs exhibit active epigenetic marks in MCF7 cells

**Figure 8:**
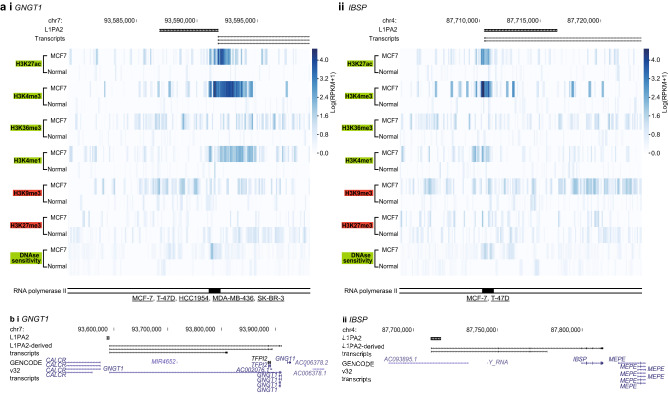
L1PA2 bearing up-regulated transcript TSSs showed cancer-specific, active epigenetic profiles. Examples are shown for (**i**) *GNGT1* and (**ii**) *IBSP*. For each gene, (**a**) histone tail modifications and DNAse sensitivity in MCF7 and normal tissues are shown for the 20 kb region centred on the L1PA2. RNA pol II binding within each L1PA2, as well as the cell lines where the binding was detected, are shown. Underlined cell line names indicate cancerous cell lines. (**b**) The exon structures of the L1PA2-derived transcripts (black), compared to the GENCODE v32 transcripts (blue), are shown^79^.

Overall, the up-regulated transcripts showed a pattern of epigenetic activation in MCF7 cells (Supplementary Fig. S8 online). We further confirmed the cancer-specific transcriptional activity of L1PA2 transposons bearing up-regulated TSSs by comparing their epigenetic states in MCF7 cells and MCF10A cells. Epigenetic profiling revealed that TSSs in L1PA2 transposons were correlated with increased active histone tail modifications and increased DNAse sensitivity in MCF7 cells, while these active marks were not observed for the same regions in MCF10A cells (Fig. 8a). These L1PA2 transposons also contained RNA pol II binding sites previously reported in breast cancer cell lines (Fig. 8a). The exon–intron structure of example L1PA2-derived transcripts, as well as the corresponding known transcripts, are shown in Fig. 8b.

## Discussion

Approximately 45% of the human genome is composed of transposons^1^. For transposons to integrate and mobilise within a host genome, by necessity they must contain cis-regulatory sequences that are compatible with host RNA polymerases and/or TFs^2–7^. This compatibility also enables the host to exapt the transposon-derived regulatory sequences to modify the transcriptional regulation of host genes, including the generation of novel transcript isoforms originating in transposon-derived promoters.

Transposons have been exapted to contribute regulatory roles in diverse biological processes, including innate immunity and pregnancy^2,4,28^. In some cases, multiple elements of a given transposon subfamily have been exapted to contribute to the co-ordinated regulation of multiple genes, such as the MER41 subfamily that contributes enhancer activities to immunity-related genes such as *AIM2*, *APOL1*, *IFI6*, and *SECTM1*^2^. By replicating highly related sequences throughout the genome, transposons provide abundant substrate for exaptation to mediate the temporal or spatial co-ordinated regulation of functionally related genes.

Individual L1PA2 transposons have been reported to modulate gene expression in various cancer types. More specifically, a hypo-methylated L1PA2 transposon is found to act as an alternate promoter to the *MET* oncogene in breast cancer, chronic myeloid leukemia and colorectal cancer, and the L1PA2*-MET* expression is associated with enhanced malignancy and poor prognosis^22–25^. In the context of breast cancer, we previously identified enriched E2F1 and MYC binding sites in the L1PA2 subfamily, and demonstrated that L1PA2 contributed the significant promoter activity for the *SYT1* oncogene in triple negative breast cancer cell lines^35^. Intrigued by these results, we hypothesised that this regulatory activity was conserved across the L1PA2 subfamily, and developed a bioinformatic pipeline for the integrated analysis of transcription factor binding and transcriptomic data to identify L1PA2-derived TF binding and promoter activity in breast cancer cells.

Here we have utilised data from the GTRD and the ChIP-Atlas databases, both of which aim to catalogue and compile an extensive range of publicly available ChIP-seq datasets^39,40^. The GTRD data was used for general analysis of TF binding, as it merges the binding sites for individual TFs into meta-clusters representing all experimental conditions (tissues, cell lines, treatments etc.), reducing the computational resources required for analysis. By contrast, the ChIP-Atlas database retains the cell line and tissue information, allowing us to assess TF binding in specific cell lines.

Our analysis revealed that when all TFs and experimental conditions in the GTRD database were considered, 54.2% of L1PA2 transposons contained at least one TFBS, and TFBSs often clustered within the 5′ UTR (Fig. 1). This represents a minimal estimation of L1PA2 regulatory potential, as many TFs have been studied under a limited number of conditions. The 5′ UTR of LINE1 transposons, approximately 900 bp in length, contains two internal promoters allowing transcription to be initiated in either direction^29,30^. This region represents a potent reservoir of regulatory activity, harbouring binding sites for a variety of TFs, including YY1, RUNX, SOX and MYC^55–58^. The LINE1 5′ UTR, particularly the antisense promoter, also contributes significant promoter activity in a variety of cell types, affecting the expression of many genes such as *SYT1*, *MET* and *JAK1*^35,59^.

To focus our analysis on the regulatory activity of L1PA2 in breast cancer, we investigated the TF binding activity of L1PA2 in MCF7 breast cancer cells. Approximately 27.9% of L1PA2 transposons were bound by at least one TF in MCF7 cells, and the binding was correlated with active epigenetic marks in the 5′ UTR, suggesting potential promoter or enhancer activities of these transposons (Fig. 2 and Supplementary Fig. S2 online). It is worth noting that L1PA2 transposons showed abundant TF binding activity in other cancer cell lines, suggesting a widespread role of the L1PA2 subfamily in modulating transcriptional regulation in cancer and potentially the rewiring of global gene expression to a state that is favourable for cancer progression (Supplementary Fig. S5). In MCF7 cells, L1PA2 transposons harboured binding sites for a total of 75 TFs. Some of these TFs have been shown to exhibit oncogenic properties in various types of cancers, such as ESR1, FOXA1 and E2F1 (Fig. 3a)^60–62^. Motif analysis revealed that the binding of these TFs was correlated with conserved sequence motifs in the 5′ UTR of L1PA2 transposons (Fig. 7). In particular, the expression of the estrogen receptor (ESR1 or ER), is the defining feature of the Luminal A and Luminal B breast cancer subtypes^48^. At least 70% of breast cancer cases are ER-positive, and ER signalling is a key driver of cancer progression and tumour growth^48,60^. Amongst all L1PA2-binding TFs in MCF7 cells, ESR1 shows the highest binding frequency, with over 80% of TF-bound L1PA2s harbouring an ESR1 binding site (Fig. 3a). The conserved ESR1 binding in L1PA2 suggests that the L1PA2 subfamily likely plays a prominent role in mediating ESR1 regulation.

In 1971, Britten and Davidson proposed that transposons could act as a vector to disperse regulatory elements and thereby recruit genes into co-regulated, co-expressed networks^27^. Intrigued by their theory, we sought to investigate whether L1PA2 transposons can act as a vector for dispersing regulatory elements that contribute to breast cancer. We analysed the frequency of co-localisation between each pair of top L1PA2-binding TFs in MCF7 cells, and found that the binding sites of these TFs indeed co-occurred significantly more frequently in L1PA2 transposons, particularly in the 5′ UTR, compared to the remainder of the genome (Figs. 3b,c, 4 and Supplementary Fig. S3 online). This enrichment of binding site co-occurrences in MCF7 cells appeared to be a distinct feature specific to the L1PA2 transposons, as other transposon subfamilies lacked or had notably fewer occurrences of TFBS co-localisation (Supplementary Fig. S4). In addition, L1PA2 transposons in the near-normal MCF10A cells also showed fewer occurrences of TF binding and TFBS co-localisation enrichment, further confirming the cancer-specific regulatory activity of this subfamily (Fig. 5). In particular, the binding sites of ESR1, which binds DNA upon estrogen stimulation, was found to co-localise with the binding sites of E2F1 and MYC, both of which play a role in transcriptional regulation of estrogen-stimulated genes^63,64^. Furthermore, MYC and CTCF also co-localised to the 5′ UTR of L1PA2 transposons, which was a pattern that has also been observed for the L1HS subfamily, and may be related to chromatin remodelling^58^. Overall, the enrichment of co-occurring TFBSs in L1PA2 transposons supported the hypothesis that L1PA2 transposons served as a vector for dispersing functionally related regulatory sequences, and thereby mediated the combinatorial control of genes in breast cancer.

Mammalian gene regulation is effected by the co-ordinated action of multiple transcription factors, acting in transcriptional complexes^47,48^. Furthermore, many TFs, such as FOXA1 and ESR1, have been shown to contribute oncogenic activity in some cancer types, while acting as tumour suppressors in other contexts, often involving different protein–protein interactions^49,50^. Therefore, understanding the activity of interacting TF networks, rather than individual TFs, often provides a more comprehensive insight into gene regulation in the cancer transcriptome. Considering only protein–protein interactions with experimental evidence in the STRING database^51^, L1PA2-binding TFs formed a highly interacting network, demonstrating that these TFs commonly interact in gene regulation (Fig. 6a). Functional enrichment analysis by ToppFun^52^ supported the oncogenic properties of these networks, as the L1PA2-binding TFs showed a statistical enrichment for transcriptional mis-regulation in multiple cancer types, including prostate cancer and breast cancer (Fig. 6b,c). Taken together, L1PA2 transposons harbour binding sites for functionally interacting, cancer-associated TFs, and contribute to co-ordinated transcriptional regulation in breast cancer.

Transcriptional regulation is often a complex and highly dynamic process, and can lead to both the activation or repression of neighbouring genes depending on the interactions of TFs. To better understand the regulatory impact of L1PA2 transposons in breast cancer, we re-analysed publicly available RNA-seq data from MCF7 breast cancer cells and MCF10A near-normal cells, and assessed the distribution of L1PA2 relative to up-regulated (activation) or down-regulated (repression) transcripts in MCF7 cells. TF binding in L1PA2 transposons was correlated with the activation of nearby transcripts (see Supplementary Fig. S6 online). Detailed assessment of TSS and L1PA2 overlap showed that the up-regulated transcripts were significantly enriched in L1PA2 transposons, and that the L1PA2 subfamily was a major driver of transcript expression in MCF7 cells (Supplementary Tables S5 and S6 online). Some of these L1PA2-derived TSSs have been annotated in the Ensembl database^65^, while many appeared to be alternative or novel TSSs that are not yet annotated (see Supplementary Table S6 online). Some L1PA2-derived transcripts shared exons with known transcripts, such as *GNGT1* and *IBSP*, representing novel isoforms (Fig. 8b). These alternate transcripts were likely products of L1PA2 epigenetic dysregulation in the cancer state, supported by increased active histone tail modifications and increased DNAse-sensitivity (Fig. 8a). Further supporting the cancer-specific regulatory activity of these L1PA2s, RNA pol II binding sites within the L1PA2s were found, and these binding sites have previously been reported, sometimes exclusively, in cancer cell lines (Fig. 8a). Amongst these L1PA2-derived up-regulated transcripts, we found the L1PA2-*MET* transcript, which had previously been found to be linked to enhanced malignancy and poor prognosis^22–25^ (see Supplementary Table S6 online). Taken together, transposons that harbour TFBSs are transcriptionally active, and provide novel TSSs to unannotated transcript isoforms in MCF7 cells. Some of these transcripts have established oncogenic properties, and it is likely that the other L1PA2-driven up-regulated transcripts, summarised in Supplementary Table S6 online, also have oncogenic functions in breast cancer.

In this study, we developed a bioinformatic pipeline for investigating the TF binding and promoter activity of transposons in specific biological contexts. Our pipeline can be applied to a broad range of transposons, cell lines and tissues types, and can also be used to study transposon-derived regulation in various disease states. In the current study, we performed an integrated analysis to assess the regulatory activity of the L1PA2 transposon subfamily in the MCF7 breast cancer cell line, and further validated the utility of our pipeline on other transposon subfamilies, as well as on leukemia and lung carcinoma cell lines. Our analyses demonstrated that L1PA2 transposons, primarily their 5′ UTR, constituted a rich reservoir of regulatory potential, as they contributed abundant functional TFBSs in various tissues and experimental conditions. In the context of breast cancer, L1PA2 transposons facilitated binding site co-localisation of functionally interacting TFs, and thereby contributed to the co-ordinated gene regulation in cancer. The transcriptional activation of L1PA2 transposons in breast cancer cells, potentially due to loosened epigenetic repression, led to alterations in the transcriptome and the production of many alternative or novel transcripts. Some of these L1PA2-driven transcripts, such as L1PA2-*MET*, have well established oncogenic properties, and it is likely that the other transcripts also exhibit similar oncogenic activities in cancer. In summary, L1PA2 transposons contribute abundant regulatory sequences for the co-ordinated gene regulation and oncogene activation in breast cancer. LINE1 transposons, including L1PA2, have been touted as potential cancer biomarkers, but have not been successfully adapted for such a use. By contrast, transcripts resulting from their transcriptional activation may serve as proxies for dysregulated transposons in tumour cells, and provide novel biomarkers for cancer detection. While further investigation is required on the function of additional putative oncogenes, these may also provide targets for therapy or disease prognosis. Furthermore, targeting L1PA2 transcriptional activation may itself provide a broad-acting target to control aberrant gene regulation in breast cancer.

## Methods

### Genomic locations of genetic entities

Genetic entities that mapped to alternate chromosomes, including TFBSs, transposons, and transcripts, were excluded from all analyses in this study. All genomic locations were in hg38 unless otherwise stated.

### Identifying TFBSs in L1PA2 and surrounding regions

TFBSs, defined as meta-clusters, were acquired from the GTRD database (version 19.10)^39^. The GTRD v19.10 database includes ChIP-seq datasets performed in 609 human cell lines and 289 tissue types, and merges binding sites identified across various experimental conditions (tissues, cell lines, treatments etc.) into meta-clusters^39^. The genomic locations (hg38) of human L1PA2 elements were retrieved from the UCSC RepeatMasker table^33,34^. For each L1PA2 element, a 20 kb region centred on the transposon was defined and divided into 100 bp bins. The genomic locations of the GTRD TFBSs were intersected with the L1PA2 elements using BedTools Intersect, where an overlap was called when at least half of a TFBS overlapped with an L1PA2 transposon, using the –f option^66^. The number of TFBSs in each 100 bp bin was counted using BedTools Coverage^66^, and normalised to the total counts of TFBSs mapped to the corresponding 20 kb regions to obtain the normalised counts. The normalised counts in each bin were averaged across all L1PA2 elements containing at least one TFBS.

### Identifying L1PA2-derived TFBSs in MCF7 breast cancer cells

All TFBSs identified in MCF7 cells were obtained from the ChIP-Atlas database (Cell Type = “MCF-7”)^40^. The ChIP-Atlas dataset used in our analysis represent TFBSs identified in MCF7 cells, by over 900 publicly available ChIP-seq experiments. These studies include experiments performed on MCF7 cells subjected to a diverse range of experimental conditions, including hormone treatments with estradiol and progesterone. The ChIP-Atlas data includes TFBSs for 100 TFs in MCF7 cells. For the purpose of consistency, we referred to the TFs by the names recorded in ChIP-Atlas, instead of converting them to the protein names (e.g. ESR1 instead of ERα). The genomic coordinates of the TFBSs were converted from hg19 to hg38 using LiftOver, and only TFBSs that were successfully converted were retained for downstream analysis^67^. The genomic locations of the TFBSs were intersected with the L1PA2 elements using BedTools Intersect, where an overlap was called when at least half of a TFBS overlapped with an L1PA2 transposon, using the –f option^66^. The number of L1PA2 elements containing at least one TFBS in MCF7 cells was counted using a custom python script, and the lengths of L1PA2 transposons were calculated from their genomic coordinates. The number of L1PA2 overlaps for each TF and the number of TFs binding to each L1PA2 were counted using a custom python script.

### Epigenetic profiling of TF-bound L1PA2 transposons in MCF7 cells and mmanormal mary tissues

L1PA2 transposons were divided into two groups based on the TF binding status in MCF7 cells. L1PA2 transposons were investigated for DNAse sensitivity, as well as active (H3K27ac, H3K4me1, H3K4me3 and H3K36me3) and repressive (H3K9me3 and H3K27me3) histone tail modifications in MCF7 cells. More specifically, epigenetic states of the 20 kb region (bin = 100 bp) centred on each L1PA2 transposon were investigated using publicly available DNAse-seq and ChIP-seq datasets, as described in Jiang and Upton^35^. Briefly, published DNAse-seq and ChIP-seq data were downloaded from ENCODE in the FASTQ format, and aligned to the human genome (hg38) using BWA mem^68,69^ (for data sources see Jiang and Upton^35^)). For each epigenetic mark, Samtools view (-c option) was used to count reads in each 100 bp bin, then values were converted to RPKM^70^. RPKM values were subsequently averaged across replicates. For ChIP-seq data, RPKM values were normalised to the input control by subtraction to produce the “normalised RPKM” values. Finally, RPKM values were averaged across L1PA2 transposons within the TF-bound or unbound group. Similarly, the histone tail modification profiles of the same regions in normal breast tissues were investigated (for data sources see Supplementary Table S7 online and Jiang and Upton^35^).

### Investigating TF co-localisation in L1PA2 transposons

To remove redundant information, overlapping L1PA2-derived binding sites of the same TF were merged to keep the most distal genomic coordinates. Locations of TFBSs in L1PA2 transposons were calculated as the distances between the middle of the binding sites and the 5′ ends of the transposons. The equivalent locations in the L1PA2 consensus sequence were then calculated by adjusting the distances by the RepStart and RepEnd information from the RepeatMasker database^33,34^. To investigate TFBS co-localisation, for each pair of L1PA2-binding TFs, the number of L1PA2 transposons containing both TFBSs was counted and the distances between the binding sites were calculated using a custom python script.

### Co-localisation enrichment in L1PA2 transposons

Genome-wide binding sites for the top binding TFs (ESR1, SFPQ, MYC, FOXA1, NR2F2, CTCF, E2F1, KDM5B, ZNF143) were identified using the MCF7 ChIP-seq data from ChIP-Atlas^40^. For each TF, non-L1PA2-derived TFBSs were identified by filtering out the previously identified L1PA2-derived TFBSs from genome-wide binding sites. Genomic locations of overlapping TFBSs were merged using BedTools Merge to remove redundant information^66^.

Here, we defined co-localised, or co-occurring TFBSs to be located within 500 bp of each other. For each TF, binding sites that co-localised with another TF were identified using BedTools Window (-w 500, -u). The proportion of co-localisation was calculated as the number of co-localising TFBSs divided by the total number of TFBSs for this TF. To account for asymmetrical co-localisation, the co-localisation proportions for each TF-TF combination were calculated with each factor as the denominator separately. For example, the proportion of ESR1 binding sites co-localising with SFPQ, and the proportion of SFPQ binding sites co-localising with ESR1, were calculated separately. The co-localisation proportions for L1PA2-derived and non-L1PA2-derived TFBSs were determined separately and compared using a one-tailed proportion z-test. To account for multiple testing, p-value < 6.2E−4 $(\frac{0.05}{{9}^{2}}$) indicated statistical significance.

### Functional annotation of TF binding in L1PA2 transposons

To estimate the interactions between these TFs, the list of TFs binding to L1PA2 transposons in MCF7 cells was used as input for the online Multiple Proteins by Names / Identifiers search in STRING v11^51^. Only protein–protein interactions with experimental evidence were considered (active interaction sources = “Experiments”, minimum required interaction score = 0.15). In order to gain further insight into the pathways or diseases the TF networks were associated with, functional enrichment analysis was performed using the ToppFun tool^52^. Bonferroni q-value < 0.05 indicated statistical significance.

### Motif analysis of TF binding in L1PA2 transposons

Binding motif position-weight matrices were obtained from the JASPAR 2020 database for ESR1 (MA0112.2), FOXA1 (MA0148.4) and E2F1 (MA0024.2)^71^. DNA sequences of L1PA2 transposons bound by each TF were extracted from the hg38 FASTA file for main chromosomes^1,72^ using BedTools Getfasta (v2.25.0) (-s option)^66^. Motif occurrences in L1PA2 sequences were identified using FIMO v5.0.5 (-norc option), with a statistical significance threshold of p-value < 1E−4^73^. Positions of the discovered motifs in the L1PA2 consensus sequence were calculated using the RepStart and RepEnd information from RepeatMasker^33^. The distribution of discovered motifs in the consensus sequence (between 0 and 6500 bp) was visualised by plotting the middle of the adjusted positions in a histogram (bin = 5 bp). For bins that contained a high number of discovered motif sequences (peaks), a sequence logo, which was a representation of sequence conservation, was generated with the motif sequences in the corresponding bins using the WebLogo online tool v2.8.2^74^. For the purpose of comparison, an L1PA2-specific sequence logo was generated for each TF using all discovered motif sequences.

### Identifying differentially expressed transcripts in MCF7 cells

SRA files of publicly available RNA-seq data in MCF7 and MCF10A cells (GSE108541, n = 3) were downloaded from the GEO database and converted to paired-end FASTQ files using command fasterq-dump –S (http://ncbi.github.io/sra-tools/)^75,76^. The quality of the sequencing data was confirmed using FastQC v0.11.8 (www.bioinformatics.babraham.ac.uk/projects/fastqc/)^77^. Reads were aligned to GENCODE v29 transcripts (hg38) using STAR 2.7.1a, with more permissive multi-mappers parameters (–outFilterMultimapNmax 100 –winAnchorMultimapNmax 200, defaults are 20 and 50 respectively)^78,79^. Transcripts were called and quantified for expression using StringTie 2.0.3 (–rf -f 0.0 -c 0.001)^54^. To include all annotated transcripts in our analysis, transcripts identified in each cell line were merged using StringTie 2.0.3 Merge^54^, and expression quantification was repeated.

Differential transcript expression analysis was performed using Ballgown 2.16.0 in R 3.6.1, which outputted the fold changes of transcripts as well as the p-values and q-values of the statistical tests^80^. In this study, differentially expressed transcripts were defined as those with a fold change of more than 2 (up-regulated), or less than 0.5 (down-regulated) in MCF7 cells relative to MCF10A cells.

We defined the TSS of a transcript to be the position at which the transcript began. L1PA2-derived transcripts were identified by intersecting the TSSs of differentially expressed transcripts with L1PA2 transposons using BedTools Intersect^66^. The exon structure and genomic locations of unknown transcripts were compared to nearby known transcripts by eye using the UCSC genome browser and information from the Ensembl database^65,72^. Unknown transcripts were considered to be associated with a known transcript if they overlapped by at least one exon.

### Enrichment analysis of differentially expressed TSSs in L1PA2 transposons

L1PA2 transposons were split into two groups based on their TF binding status. For each group, we identified the L1PA2 transposons that were located within 1 kb to 20 kb (in increments of 1 kb) away from an up-regulated or down-regulated TSS, using the BedTools Window -w option^66^. The proportions of L1PA2 transposons located nearby the TSSs were calculated by dividing the counts by the total number of L1PA2 transposons in the corresponding TF-binding group.

To test for the statistical significance of direct overlaps between TSSs and L1PA2 transposons, the expected numbers of up-regulated and down-regulated TSSs in TF-bound L1PA2 transposons were estimated by random rotation of the genome and the TSS locations (10,000 permutations), using a custom python script. The average number of overlaps was divided by the total number of TSSs for up-regulated or down-regulated transcripts to produce the expected probability of TSSs being located in L1PA2 by random chance. The observed number of TSSs overlapping L1PA2 transposons was counted with no rotation applied, and the statistical significance for enrichment or depletion was evaluated using a binomial test. P-value < 0.05 indicated statistical significance.

### Analysis of regulatory activity in non-L1PA2 transposon subfamilies and non-MCF7 cell lines

The pipeline for investigating the regulatory activity of transposons in specific biological contexts were developed in Python. As a validation of its utility, the pipeline was applied to investigate the TF binding, binding site co-localisation and promoter activity of other primate-specific LINE1 subfamilies (L1PA7, L1PA8A, L1PA10), a more distantly related LINE1 subfamily (L1M4b), and non-LINE1 subfamilies (LTR7C and LTR24B) in MCF7 cells. The genomic locations (hg38) of these transposons were retrieved from the UCSC RepeatMasker table^33,34^.

Furthermore, we investigated the regulatory activity of L1PA2 transposons in other cell lines, including the near-normal MCF10A mammary cells, A549 (lung carcinoma) and K562 (leukemia) cells. All TFBSs (hg38) identified in these cell lines were obtained from the ChIP-Atlas database^40^, and processed in the same way as the MCF7 data. The ChIP-Atlas data includes TFBSs for 192 TFs in K562 cells, 57 TFs in A549 cells and 41 TFs in MCF10A cells.

### Epigenetic profiling of up-regulated TSSs in MCF7 cells

The TSSs of up-regulated transcripts, as well as L1PA2 transposons harbouring TSSs of up-regulated transcripts were investigated for their epigenetic states and RNA pol II binding. Epigenetic states of the 20 kb region centred on each L1PA2 transposon or TSS (bin = 100 bp) in MCF7 cells were investigated using publicly available ChIP-seq and DNAse-seq datasets, as described above and in Jiang and Upton^35^). Similarly, the epigenetic profiles of the same regions in normal primary breast tissues were investigated (for data sources see Supplementary Table S7 online and Jiang and Upton^35^).

RNA pol II binding data in breast tissues and cell lines (hg19) were downloaded from ChIP-Atlas^40^ in the bed format (Cell Type Class = “Breast”), and converted to hg38 using LiftOver as previously described^67^. Overlapping binding sites were merged using BedTools Merge^66^. RNA pol II binding sites were intersected with the L1PA2 transposons using BedTools Intersect^66^, retaining information on the cell lines or tissues in which the binding sites were detected.

## Supplementary Information

Supplementary Figures.

Supplementary Table S1.

Supplementary Table S2.

Supplementary Table S3.

Supplementary Table S4.

Supplementary Table S5.

Supplementary Table S6.

Supplementary Table S7.

## Data Availability

All publicly available datasets used in this study were referenced in the Methods section. Our pipeline was developed in python, and the script can be accessed through GitHub (https://github.com/kyleupton/TEprofiler).

## References

[1] Lander ES (2001). Initial sequencing and analysis of the human genome. Nature.

[2] Chuong EB, Elde NC, Feschotte C (2016). Regulatory evolution of innate immunity through co-option of endogenous retroviruses. Science.

[3] Jordan IK, Rogozin IB, Glazko GV, Koonin EV (2003). Origin of a substantial fraction of human regulatory sequences from transposable elements. Trends Genet..

[4] Sundaram V (2014). Widespread contribution of transposable elements to the innovation of gene regulatory networks. Genome Res..

[5] Cruickshanks HA, Tufarelli C (2009). Isolation of cancer-specific chimeric transcripts induced by hypomethylation of the LINE-1 antisense promoter. Genomics.

[6] Gifford WD, Pfaff SL, Macfarlan TS (2013). Transposable elements as genetic regulatory substrates in early development. Trends Cell Biol..

[7] Chuong EB, Elde NC, Feschotte C (2017). Regulatory activities of transposable elements: From conflicts to benefits. Nat. Rev. Genet..

[8] Gerlo S, Davis JRE, Mager DL, Kooijman R (2006). Prolactin in man: A tale of two promoters. BioEssays.

[9] Flemr M (2013). A retrotransposon-driven dicer isoform directs endogenous small interfering RNA production in mouse oocytes. Cell.

[10] Notwell JH, Chung T, Heavner W, Bejerano G (2015). A family of transposable elements co-opted into developmental enhancers in the mouse neocortex. Nat. Commun..

[11] Ding Y, Berrocal A, Morita T, Longden KD, Stern DL (2016). Natural courtship song variation caused by an intronic retroelement in an ion channel gene. Nature.

[12] Guio L, Barron MG, Gonzalez J (2014). The transposable element Bari-Jheh mediates oxidative stress response in Drosophila. Mol. Ecol..

[13] McClintock B (1956). Controlling elements and the gene. Cold Spring Harb. Symp. Quant. Biol..

[14] Sharma S, Kelly TK, Jones PA (2010). Epigenetics in cancer. Carcinogenesis.

[15] Lee E (2012). Landscape of somatic retrotransposition in human cancers. Science.

[16] Choi SH (2009). Changes in DNA methylation of tandem DNA repeats are different from interspersed repeats in cancer. Int. J. Cancer.

[17] Babaian A, Mager DL (2016). Endogenous retroviral promoter exaptation in human cancer. Mob. DNA..

[18] Lamprecht B (2010). Derepression of an endogenous long terminal repeat activates the CSF1R proto-oncogene in human lymphoma. Nat. Med..

[19] Lock FE (2014). Distinct isoform of FABP7 revealed by screening for retroelement-activated genes in diffuse large B-cell lymphoma. Proc. Natl. Acad. Sci. U. S. A..

[20] Steidl C (2012). Gene expression profiling of microdissected Hodgkin Reed-Sternberg cells correlates with treatment outcome in classical Hodgkin lymphoma. Blood.

[21] Wolff EM (2010). Hypomethylation of a LINE-1 promoter activates an alternate transcript of the MET oncogene in bladders with cancer. PLoS Genet..

[22] Roman-Gomez J (2005). Promoter hypomethylation of the LINE-1 retrotransposable elements activates sense/antisense transcription and marks the progression of chronic myeloid leukemia. Oncogene.

[23] Weber B, Kimhi S, Howard G, Eden A, Lyko F (2010). Demethylation of a LINE-1 antisense promoter in the cMet locus impairs Met signalling through induction of illegitimate transcription. Oncogene.

[24] Hur K (2014). Hypomethylation of long interspersed nuclear element-1 (LINE-1) leads to activation of proto-oncogenes in human colorectal cancer metastasis. Gut.

[25] Miglio U (2018). The expression of LINE1-MET chimeric transcript identifies a subgroup of aggressive breast cancers. Int. J. Cancer.

[26] Anwar SL, Wulaningsih W, Lehmann U (2017). Transposable elements in human cancer: Causes and consequences of deregulation. Int. J. Mol. Sci..

[27] Britten RJ, Davidson EH (1971). Repetitive and non-repetitive DNA sequences and a speculation on the origins of evolutionary novelty. Q. Rev. Biol..

[28] Lynch VJ, Leclerc RD, May G, Wagner GP (2011). Transposon-mediated rewiring of gene regulatory networks contributed to the evolution of pregnancy in mammals. Nat. Genet..

[29] Speek M (2001). Antisense promoter of human L1 retrotransposon drives transcription of adjacent cellular genes. Mol. Cell. Biol..

[30] Swergold GD (1990). Identification, characterization, and cell specificity of a human LINE-1 promoter. Mol. Cell. Biol..

[31] Boissinot S, Furano AV (2001). Adaptive evolution in LINE-1 retrotransposons. Mol. Biol. Evol..

[32] Ovchinnikov I, Rubin A, Swergold GD (2002). Tracing the LINEs of human evolution. Proc. Natl. Acad. Sci. U. S. A..

[33] Smit, A., Hubley, R & Green, P. *RepeatMasker Open-4.0.* http://www.repeatmasker.org (2013–2015).

[34] Karolchik D (2004). The UCSC Table Browser data retrieval tool. Nucleic Acids Res..

[35] Jiang J-C, Upton KR (2019). Human transposons are an abundant supply of transcription factor binding sites and promoter activities in breast cancer cell lines. Mob. DNA..

[36] Jang HS (2019). Transposable elements drive widespread expression of oncogenes in human cancers. Nat. Genet..

[37] Chen H (2015). An integrative analysis of TFBS-clustered regions reveals new transcriptional regulation models on the accessible chromatin landscape. Sci. Rep..

[38] Sexton CE, Han MV (2019). Paired-end mappability of transposable elements in the human genome. Mob. DNA..

[39] Yevshin I, Sharipov R, Kolmykov S, Kondrakhin Y, Kolpakov F (2019). GTRD: A database on gene transcription regulation-2019 update. Nucleic Acids Res..

[40] Oki S (2018). ChIP-Atlas: A data-mining suite powered by full integration of public ChIP-seq data. EMBO Rep..

[41] Meyer CA, Liu XS (2014). Identifying and mitigating bias in next-generation sequencing methods for chromatin biology. Nat. Rev. Genet..

[42] Tsompana M, Buck MJ (2014). Chromatin accessibility: a window into the genome. Epigenet. Chromatin.

[43] Zhang W (2012). High-resolution mapping of open chromatin in the rice genome. Genome Res..

[44] Barski A (2007). High-resolution profiling of histone methylations in the human genome. Cell.

[45] Creyghton MP (2010). Histone H3K27ac separates active from poised enhancers and predicts developmental state. Proc. Natl. Acad. Sci. U. S. A..

[46] Peters AH (2003). Partitioning and plasticity of repressive histone methylation states in mammalian chromatin. Mol. Cell.

[47] Wasserman WW, Sandelin A (2004). Applied bioinformatics for the identification of regulatory elements. Nat. Rev. Genet..

[48] Harbeck N (2019). Breast cancer. Nat. Rev. Dis. Primers.

[49] Liu Y, Ma H, Yao J (2020). ERα, a key target for cancer therapy: A review. Oncol. Targets Ther.

[50] Park YL (2019). Forkhead-box A1 regulates tumor cell growth and predicts prognosis in colorectal cancer. Int. J. Oncol..

[51] Szklarczyk D (2019). STRING v11: Protein-protein association networks with increased coverage, supporting functional discovery in genome-wide experimental datasets. Nucleic Acids Res..

[52] Chen J, Bardes EE, Aronow BJ, Jegga AG (2009). ToppGene Suite for gene list enrichment analysis and candidate gene prioritization. Nucleic Acids Res..

[53] Vernimmen D, Bickmore WA (2015). The Hierarchy of transcriptional activation: From enhancer to promoter. Trends Genet..

[54] Pertea M (2015). StringTie enables improved reconstruction of a transcriptome from RNA-seq reads. Nat. Biotechnol..

[55] Becker KG, Swergold G, Ozato K, Thayer RE (1993). Binding of the ubiquitous nuclear transcription factor YY1 to a cis regulatory sequence in the human LINE-1 transposable element. Hum. Mol. Genet..

[56] Yang N, Zhang L, Zhang Y, Kazazian HH (2003). An important role for RUNX3 in human L1 transcription and retrotransposition. Nucleic Acids Res..

[57] Tchénio T, Casella JF, Heidmann T (2000). Members of the SRY family regulate the human LINE retrotransposons. Nucleic Acids Res..

[58] Sun X (2018). Transcription factor profiling reveals molecular choreography and key regulators of human retrotransposon expression. Proc. Natl. Acad. Sci. U. S. A..

[59] Criscione SW (2016). Genome-wide characterization of human L1 antisense promoter-driven transcripts. BMC Genom..

[60] Lei JT, Gou X, Seker S, Ellis MJ (2019). ESR1 alterations and metastasis in estrogen receptor positive breast cancer. J. Cancer Metastasis Treat..

[61] Zacharatos P (2004). Distinct expression patterns of the transcription factor E2F–1 in relation to tumour growth parameters in common human carcinomas. J. Pathol..

[62] Horiuchi D (2012). MYC pathway activation in triple-negative breast cancer is synthetic lethal with CDK inhibition. J. Exp. Med..

[63] Stender JD (2007). Estrogen-regulated gene networks in human breast cancer cells: Involvement of E2F1 in the regulation of cell proliferation. Mol. Endocrinol..

[64] Cheng AS (2006). Combinatorial analysis of transcription factor partners reveals recruitment of c-MYC to estrogen receptor-alpha responsive promoters. Mol. Cell.

[65] Aken BL (2016). The Ensembl gene annotation system. Database.

[66] Quinlan AR, Hall IM (2010). BEDTools: A flexible suite of utilities for comparing genomic features. Bioinformatics.

[67] Hinrichs AS (2006). The UCSC Genome Browser Database: Update 2006. Nucleic Acids Res..

[68] Li H, Durbin R (2009). Fast and accurate short read alignment with Burrows-Wheeler transform. Bioinformatics.

[69] ENCODE Project Consortium (2012). An integrated encyclopedia of DNA elements in the human genome. Nature.

[70] Li H (2009). The sequence alignment/map format and SAMtools. Bioinformatics.

[71] Fornes O (2019). JASPAR 2020: Update of the open-access database of transcription factor binding profiles. Nucleic Acids Res..

[72] Kent WJ (2002). The human genome browser at UCSC. Genome Res..

[73] Grant CE, Bailey TL, Noble WS (2011). FIMO: scanning for occurrences of a given motif. Bioinformatics.

[74] Crooks GE, Hon G, Chandonia JM, Brenner SE (2004). WebLogo: a sequence logo generator. Genome Res..

[75] SRA Toolkit Development Team. http://ncbi.github.io/sra-tools/.

[76] Barrett T (2013). NCBI GEO: Archive for functional genomics data sets—update. Nucleic Acids Res..

[77] Andrews, S. *FastQC: A Quality Control Tool for High Throughput Sequence Data.* https://www.bioinformatics.babraham.ac.uk/projects/fastqc/ (2010).

[78] Dobin A (2013). STAR: ultrafast universal RNA-seq aligner. Bioinformatics.

[79] Frankish A (2019). GENCODE reference annotation for the human and mouse genomes. Nucleic Acids Res..

[80] Frazee AC (2015). Ballgown bridges the gap between transcriptome assembly and expression analysis. Nat. Biotechnol..

