# Supplementary material for: Integrated transcription factor profiling with transcriptome analysis identifies L1PA2 transposons as global regulatory modulators in a breast cancer model
Source: Sci Rep. 2021 Apr 13;11:8083. doi: 10.1038/s41598-021-86395-9 (PMC8044218; doi:10.1038/s41598-021-86395-9)
Supplement: Supplementary file 1 — Supplementary Figures. [file 41598_2021_86395_MOESM1_ESM.pdf]

SUPPLEMENTARY INFORMATION

Integrated transcription factor profiling with transcriptome analysis identifies L1PA2 transposons as global regulatory modulators in a breast cancer model

Jiayue-Clara Jiang[1], Joseph A. Rothnagel[1], Kyle Upton[1*]

[1]School of Chemistry and Molecular Biosciences, The University of Queensland, St Lucia, QLD 4072, Australia

Corresponding author:

*Kyle Upton: k.upton@uq.edu.au

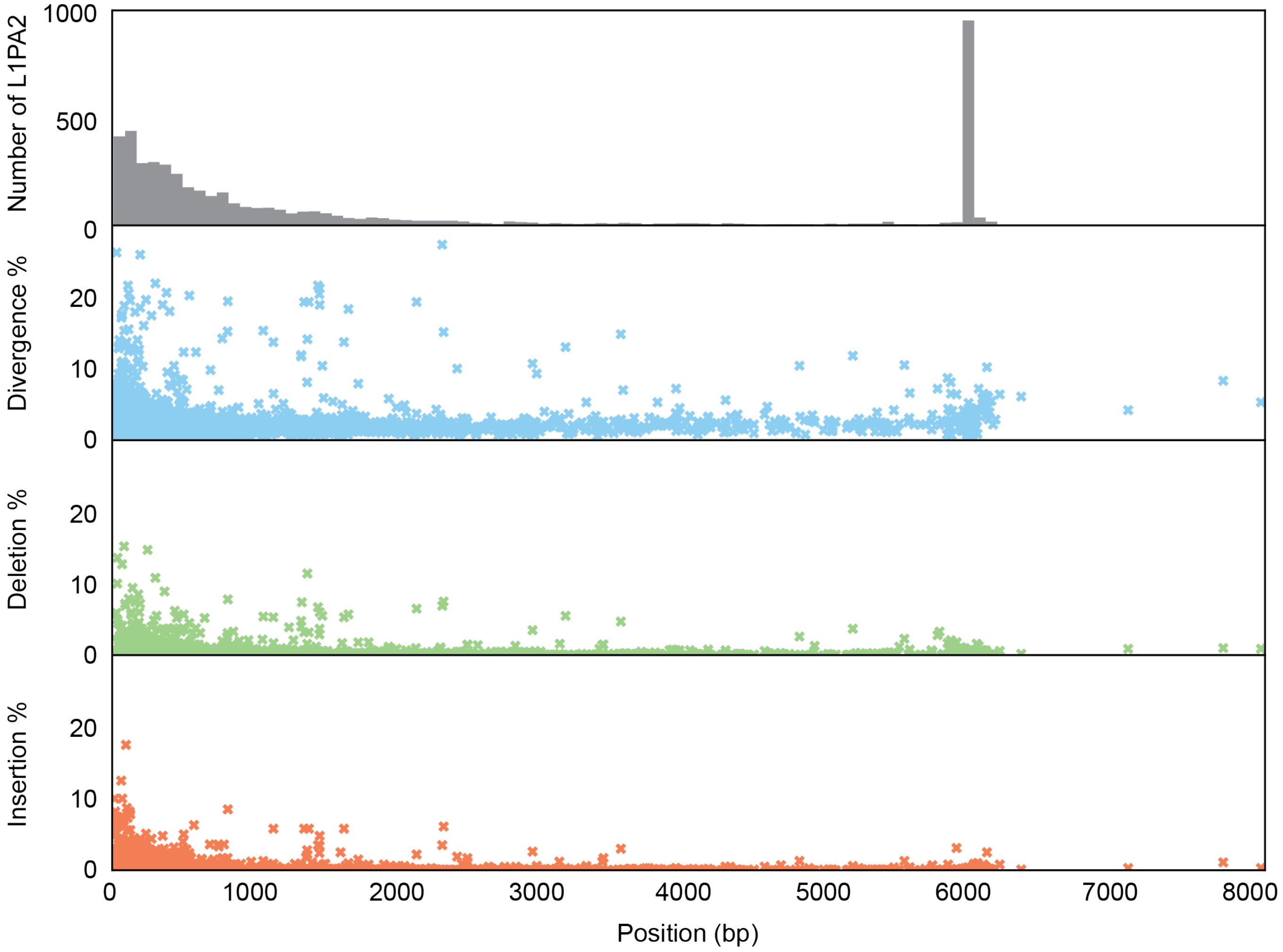

Supplementary Figure S1. Human L1PA2 structural summary. According to RepeatMasker annotations (Karolchik et al. 2004; Smit 2013-2015), there are approximately 4,940 copies of L1PA2 transposons in the human genome. Top panel displays the length distribution of L1PA2 transposons. The bottom three panels display the percentages of divergence, deletion and insertion against the length of each human L1PA2. Length, degree of divergence, deletion and insertion were calculated using the "genoStart", "genoEnd", "milliDev", "milliDel" and "milliIns" columns from the UCSC RepeatMasker table (Karolchik et al. 2004; Smit 2013-2015).

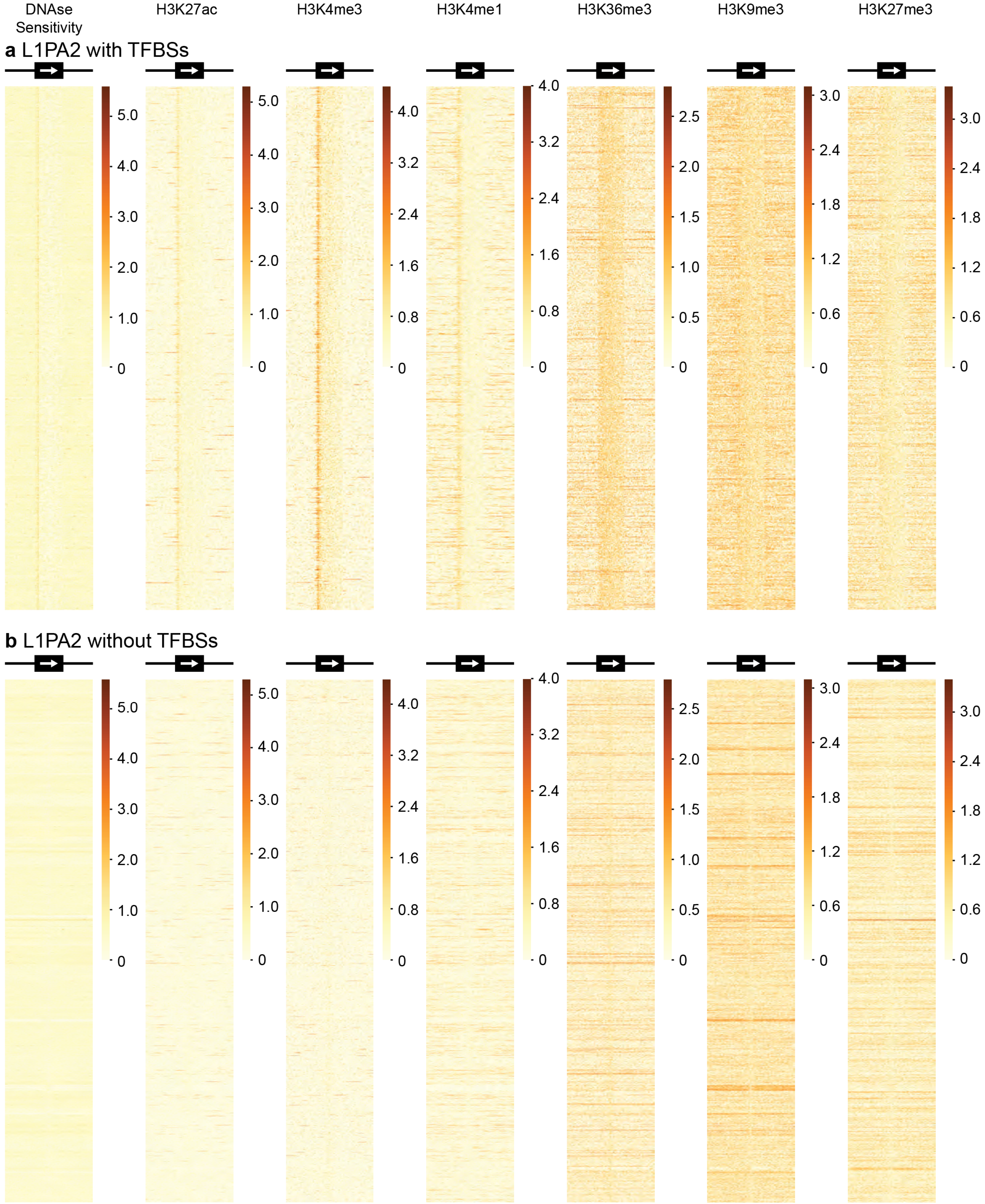

Supplementary Figure S2. TF binding in L1PA2 was correlated with DNAse sensitivity and active histone tail modifications in MCF7 cells. The RPKM values of the DNAse-seq data and the RPKM values of histone modification ChIP-seq data are transformed (n^0.25) and shown in heatmaps for the 20kb region (bin = 100 bp) centred on L1PA2 transposons **a**) with, and **b**) without TFBSs. The rectangle and arrow indicate the position and orientation (5′ to 3′) of L1PA2 transposons respectively.

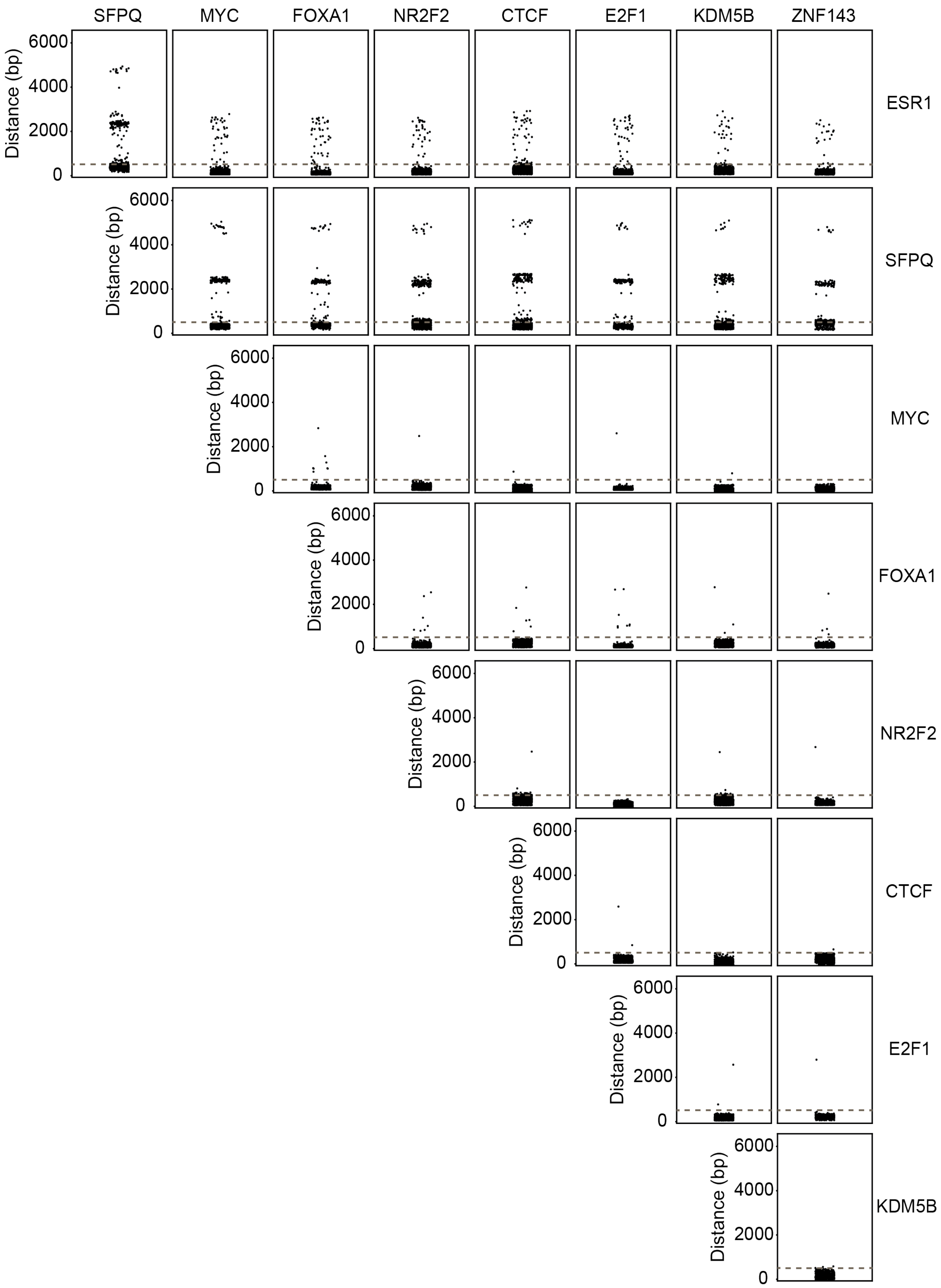

Supplementary Figure S3. The majority of TFBSs are located in close proximity within L1PA2s. Pairwise strip plots indicate the distances (bp) between the binding sites of each pair of TFs found within the same L1PA2 transposons. The grey line indicates 500 bp, a threshold selected for defining TFBS co-localisation.

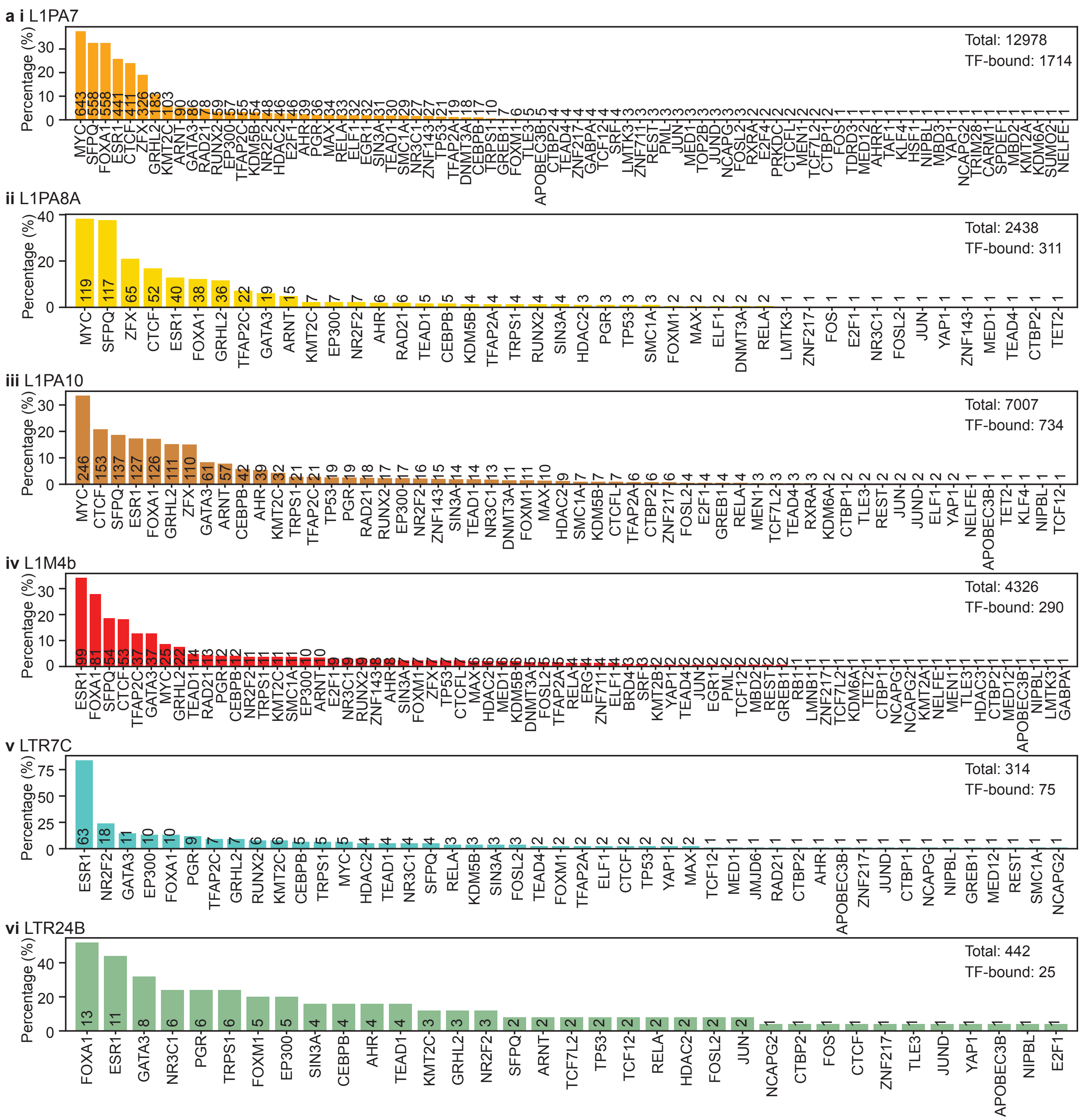

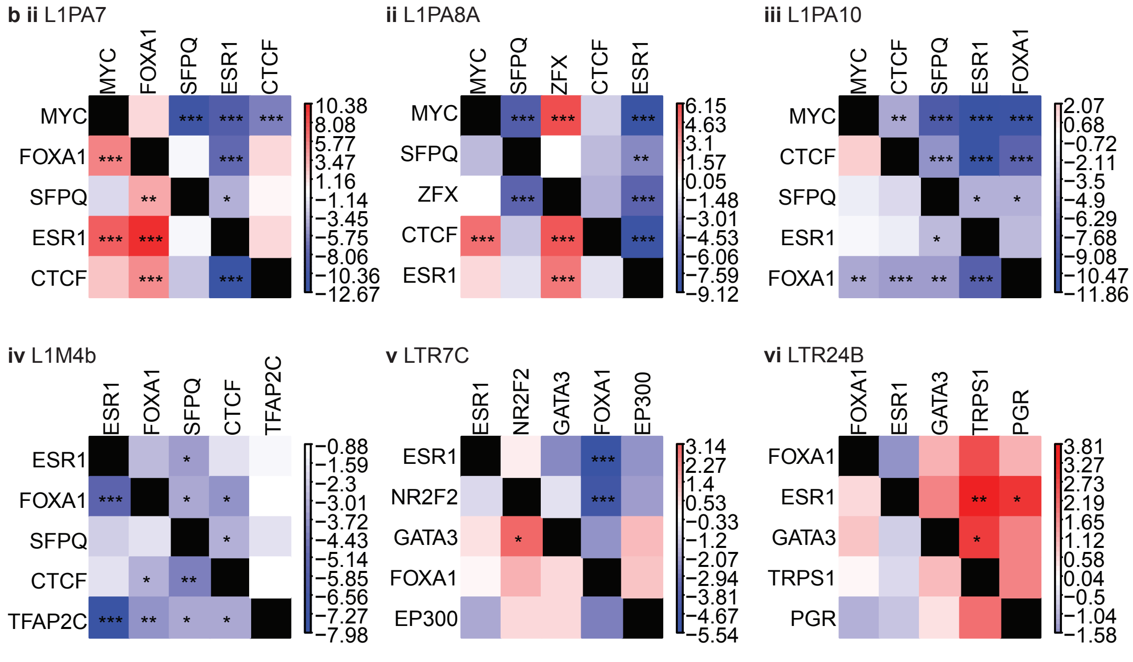

Supplementary Figure S4. TF binding profiles of non-L1PA2 transposon subfamilies in MCF7 cells. **a)** The frequency of TF binding in each subfamily. For each subfamily, bars and numbers show the percentages and counts of TF-bound transposons that harboured binding sites of each TF respectively. The numbers of total and TF-bound transposons in each subfamily are shown. **b)** TF binding site co-localisation for frequently binding TFs. For each TF-TF combination, the colocalisation proportions were compared (transposon versus non-transposon) using a one-tailed proportion z-test. The heatmap indicates the z-scores of the proportion tests, where a positive z-score (red) indicates more co-localisation in transposons, and a negative z-score (blue) indicates more co-localisation in the rest of the genome. $P < 0.002$ (=0.05/25): *, $p < 0.0001$: **, $p < 0.00001$: ***. To account for asymmetrical co-localisation, the proportions and z-scores correspond to the chances of the TF in the row co-localising with the TF in the column.

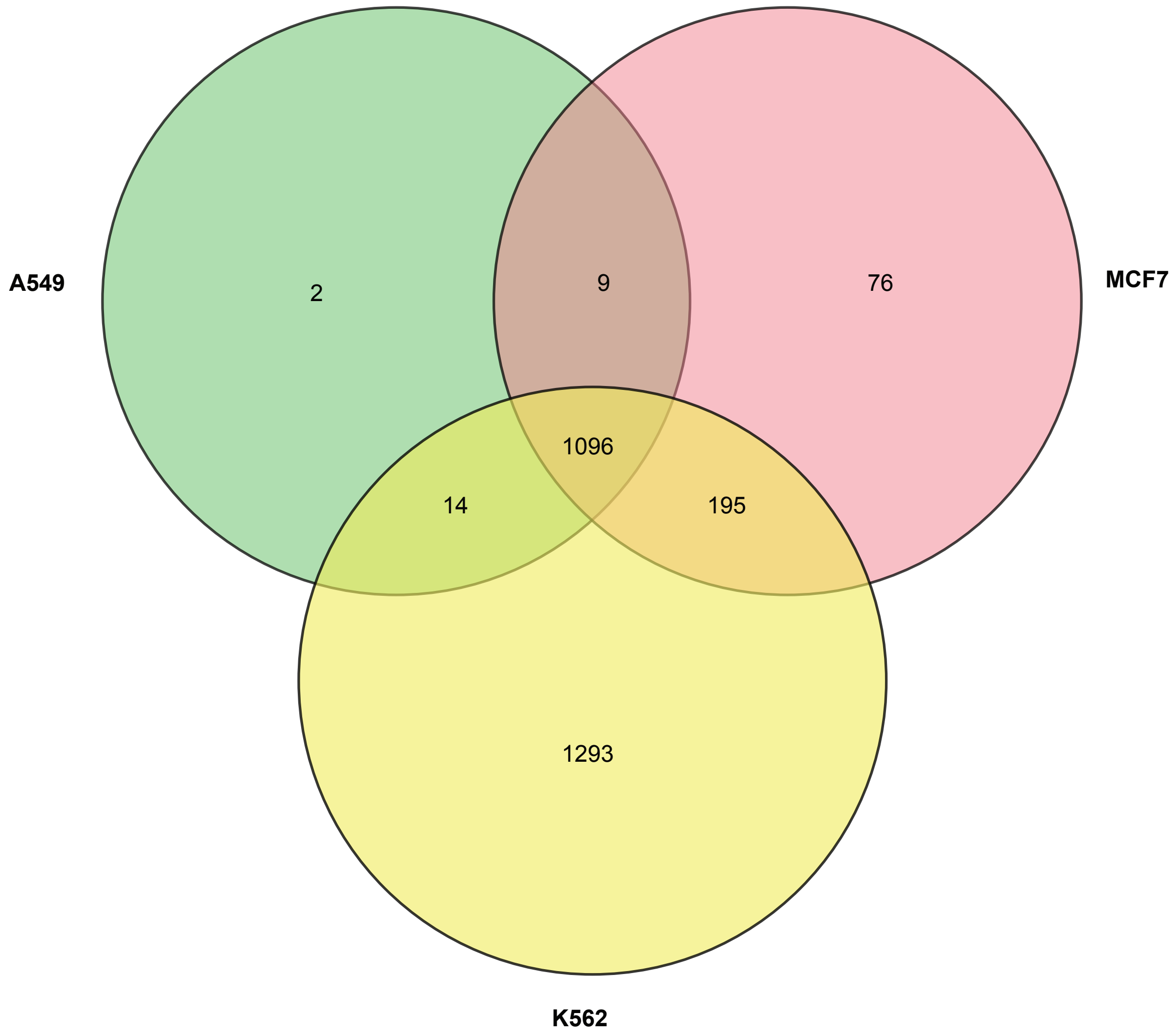

Supplementary Figure S5. Venn diagram showing overlaps between L1PA2 transposons that harboured TFBSs in MCF7, K562 and A549 cells (not drawn to scale).

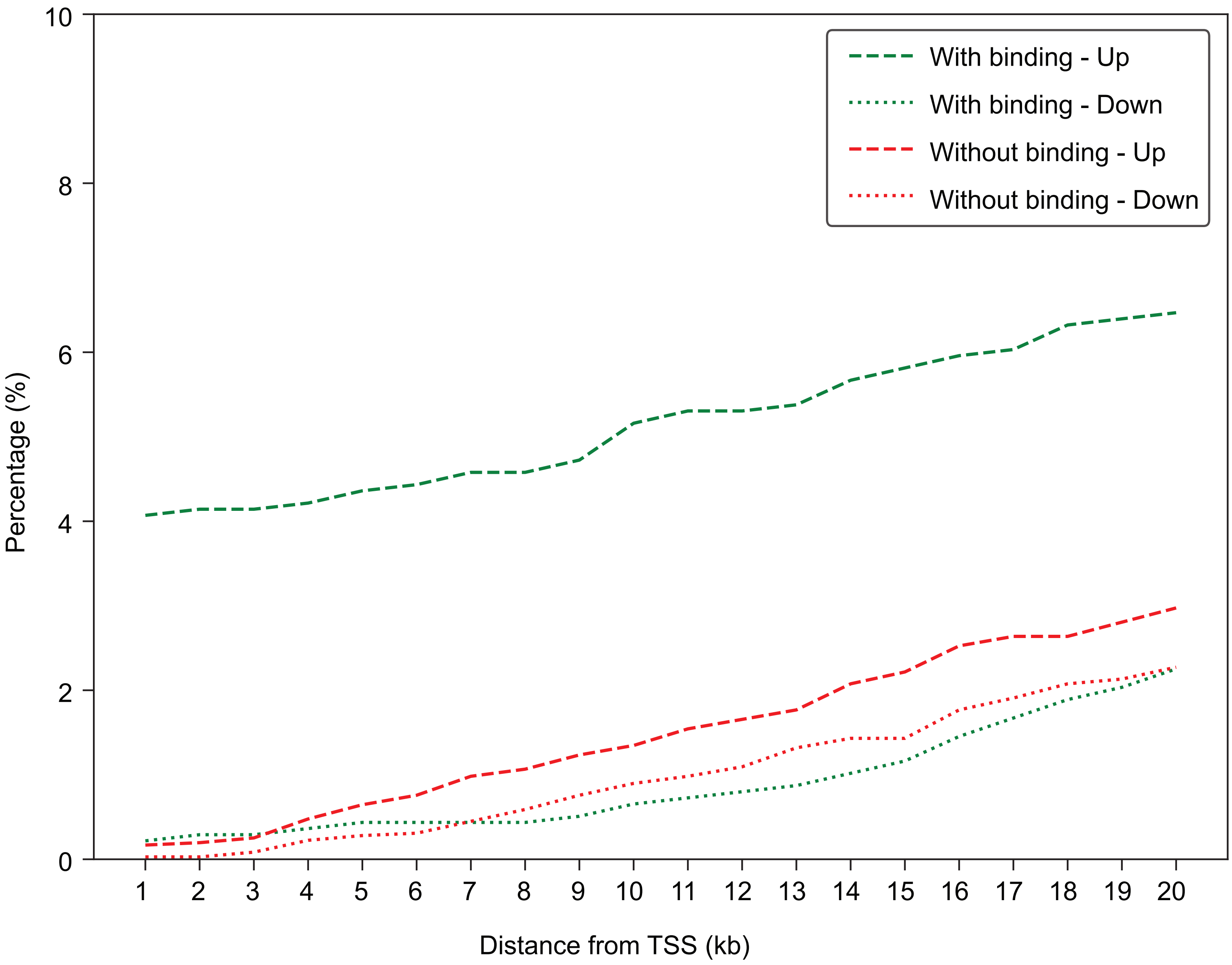

Supplementary Figure S6. TF binding in L1PA2 transposons was correlated with activation of neighbouring genes. The percentages of transposons located up to 20 kb away from the TSSs of differentially expressed transcripts are shown for L1PA2 transposons with (green) and without (red) binding. Dash and dotted lines indicated up-regulated and down-regulated transcripts respectively.

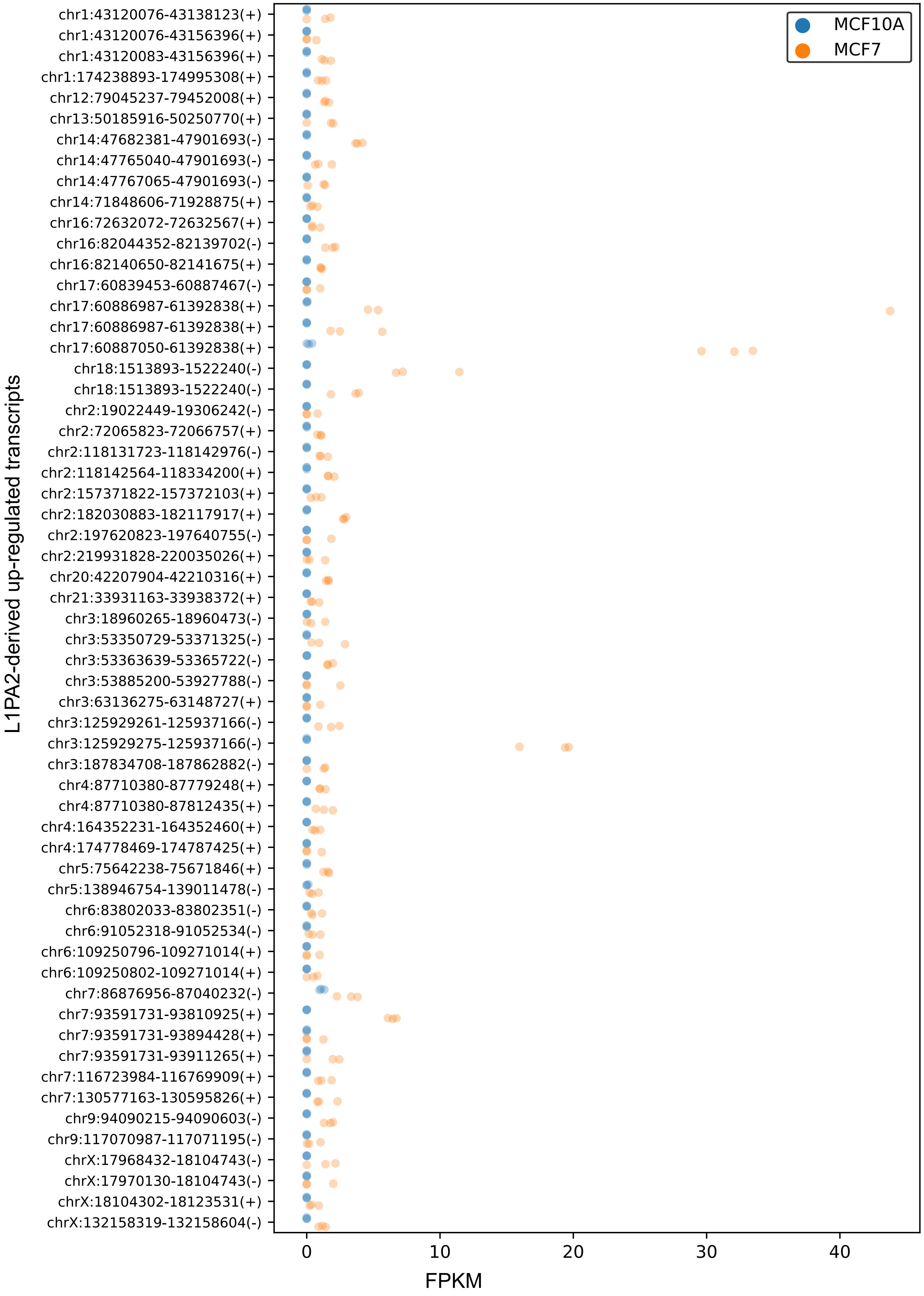

Supplementary Figure S7. Transcript expression level of up-regulated L1PA2-derived transcripts in MCF7 (orange) and MCF10A (blue) cells, as quantified by Stringtie. Each transcript is annotated with their genome location.

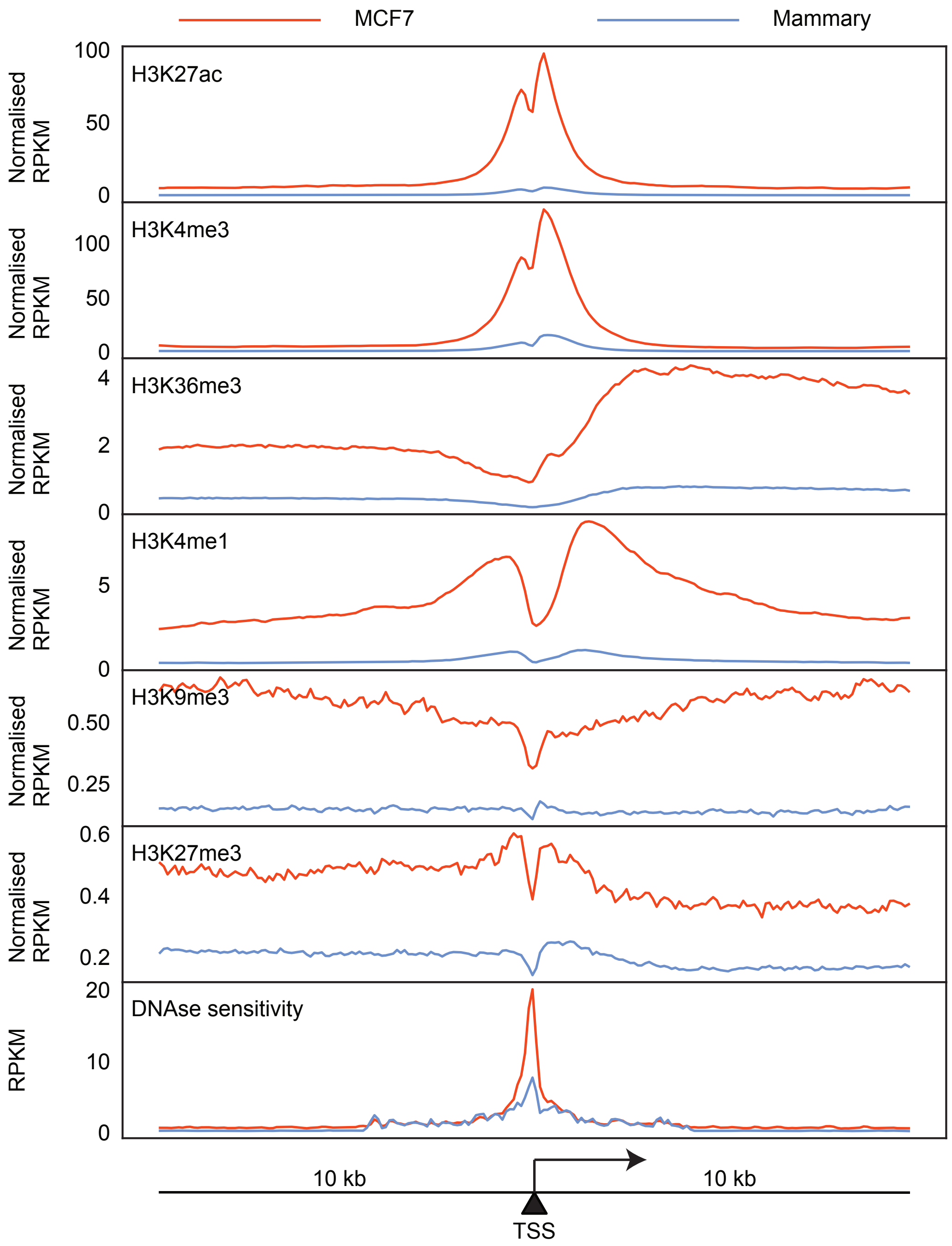

Supplementary Figure S8. The TSSs of up-regulated transcript (n = 9,375) showed cancer-specific, active epigenetic profiles. The average normalised RPKM values of histone tail modifications and the average RPKM values of DNAse sensitivity in MCF7 (orange) and normal tissues (blue) are shown for the 20 kb region centred on the TSSs. The black arrow indicates the orientation of transcription.
